# Vitamin D and Health Outcomes: State-of-the-Art Review of Triangulated Evidence and Ongoing Controversies

**DOI:** 10.1007/s13668-026-00748-2

**Published:** 2026-03-18

**Authors:** Maria Dalamaga, Rodopi Emfietzoglou, Dimitra Petropoulou, Maria Kypraiou, Dimitris C. Kounatidis, Natalia G. Vallianou, Spyridon Karras, Faidon Magkos, Irene Karampela

**Affiliations:** 1 Department of Biological Chemistry, Medical School, National and Kapodistrian University of Athens, Mikras Asias 75, Athens, 11527 Greece; 2 Assisting Nature Centre of Reproduction and Genetics, Thessaloniki, 57001 Greece; 3 Diabetes Center, First Propaedeutic Department of Internal Medicine, Medical School, National and Kapodistrian University of Athens, Laiko General Hospital, Athens, 11527 Greece; 4 First Department of Internal Medicine, Sismanogleio General Hospital, Athens, 15126 Greece; 5 Laboratory of Biological Chemistry, Medical School, Aristotle University, Thessaloniki, 55535 Greece; 6 Department of Nutrition, Exercise and Sports, University of Copenhagen, Rolighedsvej 26, Frederiksberg C, 1958 Denmark; 7 Second Department of Critical Care, Attikon General University Hospital, National and Kapodistrian University of Athens, Athens, 12462 Greece

**Keywords:** 25(OH)D, Autoimmune disorders, Calcitriol, Cancer, Cardiovascular disease, COVID-19, Diabetes, Infection, Mendelian randomization, Mortality, Osteoporosis, Randomized controlled trial, Vitamin D

## Abstract

**Purpose of Review:**

Vitamin D is a pleiotropic hormone with an established role in skeletal integrity and broader actions in immune regulation, inflammation, cellular proliferation, and energy homeostasis. Despite decades of research, its extra-skeletal effects remain controversial, largely due to discordant findings across observational studies, Mendelian randomization studies (MRS), and randomized controlled trials (RCTs). Unlike many prior reviews, this state-of-the-art review synthesizes triangulated evidence across these study designs to clarify outcome-specific causal relationships and ongoing controversies.

**Recent Findings:**

Triangulated evidence provides strong and consistent support for a causal role of vitamin D in skeletal health, particularly in the prevention and treatment of rickets and osteomalacia, and in fracture risk reduction among vitamin D–deficient and older populations. For selected extra-skeletal outcomes, modest and threshold-dependent benefits are observed, including reductions in cancer mortality, protection against autoimmune disorders, most convincingly multiple sclerosis, and decreased risk of acute respiratory infections, including COVID-19, primarily in individuals with low baseline 25(OH)D concentrations. In contrast, associations with cardiovascular disease, metabolic disorders, obesity, and most neuropsychiatric outcomes are not consistently supported by genetic or interventional evidence, suggesting limited or non-causal effects. Across outcomes, evidence indicates a non-linear relationship between vitamin D status and health, with increased risk concentrated at low 25-hydroxyvitamin D concentrations and limited benefit beyond sufficiency. All-cause mortality shows a modest, threshold-dependent association, with supplementation benefits largely confined to deficient or older populations. Key challenges include assay variability, non-linear dose–response relationships, and RCT designs that frequently enroll vitamin D–replete populations, resulting in substantial methodological heterogeneity and limiting causal inference.

**Summary:**

Overall, the presented triangulated model may reconcile longstanding inconsistencies by reframing vitamin D as a context-dependent determinant of health. These findings argue against indiscriminate population-wide supplementation and support targeted strategies focused on the identification and correction of deficiency. Vitamin D should be regarded neither as a universal panacea nor as a trivial supplement, but as a context-dependent hormone whose clinical value lies in outcome-specific correction of deficiency.

**Graphical Abstract:**

Created in BioRender by Dimitra Petropoulou (January 20, 2026) BioRender.com/rd3udxs.

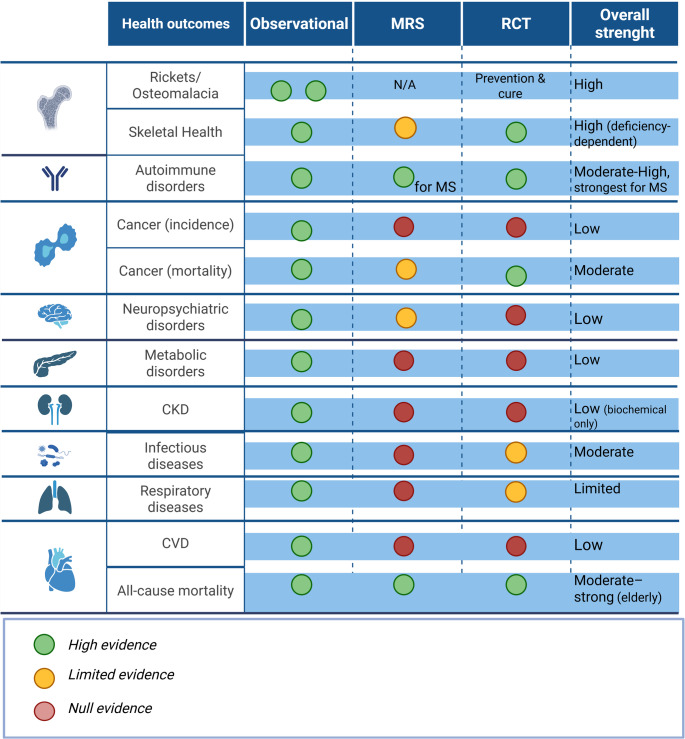

## Introduction

Vitamin D is essential for the regulation of calcium and phosphate homeostasis, with a well-established role in skeletal health, including bone mineralization and prevention of rickets and osteomalacia [1]. It is primarily synthesized in the skin through ultraviolet B (UVB) exposure, with limited dietary contributions from oily fish, eggs, and fortified foods [1]. Following cutaneous synthesis or dietary intake, vitamin D undergoes hepatic conversion to 25-hydroxyvitamin D (25(OH)D), the main circulating biomarker of vitamin D status, and subsequent renal or extra-renal hydroxylation to the biologically active metabolite 1,25-dihydroxyvitamin D [2]. Vitamin D is not considered a true vitamin but a prohormone, as endogenous synthesis under adequate UVB exposure can meet physiological requirements [3].

Interindividual variability in circulating 25(OH)D concentrations is substantial and reflects both environmental and genetic determinants. Common genetic variation in vitamin D–pathway genes, including *GC*, *CYP2R1*, and *DHCR7/NADSYN1*, contributes to interindividual differences in circulating 25(OH)D concentrations, as consistently demonstrated in large genome-wide association studies (GWAS) [4, 5].

Beyond musculoskeletal effects, vitamin D exhibits pleiotropic functions, influencing immune modulation, inflammation, cellular proliferation, differentiation, and energy homeostasis [2]. Peripheral tissues, including immune cells, express the vitamin D receptor (VDR) and possess the enzymatic machinery required for local activation of vitamin D, supporting autocrine and paracrine actions [2]. Mechanistically, vitamin D signaling modulates cytokine production, antimicrobial peptide synthesis, epigenetic regulation, and components of the renin–angiotensin system, providing biologically plausible links to cardiometabolic, autoimmune, infectious, and neoplastic diseases [6].

Observational studies have consistently reported associations between low serum 25(OH)D concentrations and increased risks of cardiovascular disease (CVD), diabetes, cancer, autoimmune disorders, respiratory infections, adverse pregnancy outcomes, and all-cause mortality [2, 3]. However, the causal interpretation of these associations remains controversial, as randomized controlled trials (RCTs) and Mendelian randomization studies (MRS) have frequently failed to demonstrate broad benefits of vitamin D supplementation in non-deficient populations [7, 8]. These discrepancies raise concerns about residual confounding, reverse causation, and threshold-dependent effects.

Hypovitaminosis D is a global public health concern, although reported prevalence varies widely by population, latitude, season, ethnicity, and assay methodology [9–13]. A recent global analysis estimated that approximately 48% of adults have serum 25(OH)D concentrations below 50 nmol/L, with severe deficiency (< 25 nmol/L) affecting a smaller but clinically relevant proportion [14]. High-risk groups include older adults, individuals with obesity, darker skin pigmentation, limited sun exposure, and those with chronic kidney or liver disease or malabsorptive disorders [12, 15]. Public health strategies such as food fortification have successfully reduced deficiency rates in selected populations, notably in Finland [9, 10].

Controversy persists regarding optimal serum 25(OH)D thresholds, screening strategies, and supplementation regimens. Major organizations, including the Endocrine Society (ES) and the National Academy of Medicine (NAM), differ in their definitions of deficiency and sufficiency, reflecting divergent weighting of observational, mechanistic, and trial-based evidence [1, 3, 16]. Moreover, non-linear and U-shaped associations between 25(OH)D levels and selected outcomes further complicate interpretation, underscoring the need for caution at both very low and very high concentrations [17, 18].

Despite decades of research, uncertainty persists regarding which associations between vitamin D and disease reflect true causality. Previous reviews have summarized individual aspects of vitamin D physiology or clinical outcomes; however, none have attempted to reconcile conflicting evidence across mechanistic, genetic, observational and interventional data. Triangulation refers to the integration of evidence derived from multiple, methodologically distinct study designs to strengthen causal inference. In nutritional and clinical epidemiology, it combines results from observational cohorts (which reveal associations), MRS (which assess genetic causality), and RCTs (which provide interventional validation). Consistent results across methods strengthen causal inference, while discrepancies may show confounding, threshold-effects or reverse causation.

Therefore, to address this gap, the present review applies a triangulated evidence model to evaluate causal and threshold-dependent associations between vitamin D status and major health outcomes, while highlighting persistent controversies and areas of clinical uncertainty. In this review, triangulation is operationalized using predefined interpretative criteria. Outcomes are considered to show strong causal support when concordant findings are observed across observational studies, MRS and RCTs. When observational associations are not supported by genetic or interventional evidence, findings are interpreted cautiously as potentially non-causal or reflective of residual confounding. When effects are confined to individuals with low baseline 25(OH)D concentrations, they are interpreted as threshold-dependent rather than universal. These criteria are applied consistently across health outcome sections to enhance coherence and transparency of inference.

## Literature Search

A comprehensive literature search was conducted in PubMed/MEDLINE, Scopus, and Web of Science up to December 2025 using combinations of the following terms: “vitamin D,” “25-hydroxyvitamin D,” “deficiency,” “randomized controlled trial,” “meta-analysis,” “systematic review,” “Mendelian randomization studies”, “mortality,” “cardiovascular,” “cancer,” “diabetes,” “obesity,” “autoimmune disorders,” “COVID-19”, “long COVID”, “respiratory infections”, “skeletal disorders”, etc. Priority was given to meta-analyses, systematic reviews, MRS, and RCTs in adult human populations. No language or geographic restrictions were applied, but English-language publications were prioritized. Reference lists of key articles and relevant guidelines were manually screened to identify additional sources. Evidence quality and methodological rigor were assessed through consideration of study design, population size, vitamin D thresholds or dosing regimens and outcome validity. The review focuses on disease-specific evidence across major health outcomes, with emphasis on areas of agreement, inconsistency, and unresolved controversies. Finally, we acknowledge that the body of literature on vitamin D is extensive, and while we aimed to incorporate the highest levels of evidence, not all available publications could be discussed in detail within this review.

## Brief Summary of Vitamin D Metabolism and Mechanisms of Action

Vitamin D is a secosteroid prohormone obtained through endogenous synthesis in the skin upon UVB exposure (vitamin D₃) and, to a lesser extent, from dietary sources such as oily fish, eggs, fortified foods, or supplements (vitamin D₂/D₃) [19]. Vitamin D₃ (cholecalciferol) and vitamin D₂ (ergocalciferol) undergo similar initial metabolic activation via hepatic 25-hydroxylation and renal 1α-hydroxylation, but differ in their subsequent metabolism, potency, and pharmacokinetics [20].

**Fig. 1:**
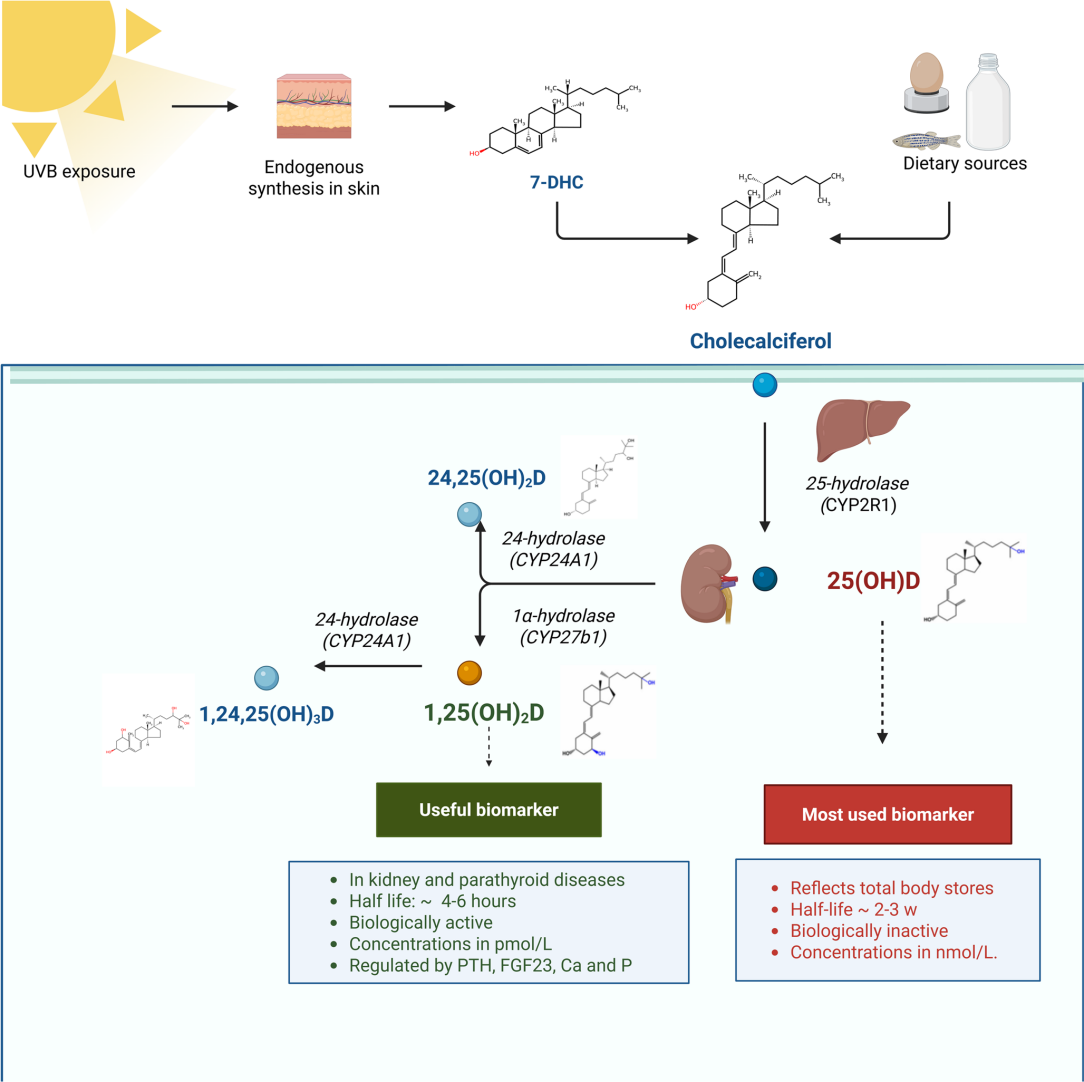
Biochemical pathways of vitamin D metabolism. *Abbreviations*: 7-DHC, 7-dehydrocholesterol; CA, calcium; FGF23; fibroblast growth factor 23; P, phosphate; PTH, parathyroid hormone. Created in BioRender by Dimitra Petropoulou (January 20, 2026) BioRender.com/9xf2d08

As depicted in Fig. 1, cutaneous synthesis begins from 7-dehydrocholesterol (7-DHC), whose availability is regulated by 7-DHC reductase (DHCR7), thereby linking cholesterol to vitamin D synthesis [21]. Following cutaneous synthesis or intestinal absorption, vitamin D is transported in the circulation bound primarily to vitamin D–binding protein (VDBP). The first hydroxylation occurs in the liver, mainly via CYP2R1, producing 25-hydroxyvitamin D (25(OH)D), the principal circulating biomarker of vitamin D status, with a serum half-life of approximately 2–3 weeks [20]. This step is weakly regulated and reflects both endogenous synthesis and dietary intake. Recent evidence indicates that hepatic CYP2R1 expression is downregulated in obesity and diabetes, contributing to lower circulating 25(OH)D concentrations despite adequate intake [22].

The second hydroxylation is catalyzed by 1α-hydroxylase (CYP27B1), predominantly in the kidney, generating the biologically active hormone 1,25-dihydroxyvitamin D (1,25(OH)₂D, calcitriol) [25]. Importantly, extra-renal expression of CYP27B1 in immune and epithelial cells enables local autocrine and paracrine production of calcitriol, functionally linking vitamin D metabolism to immune and inflammatory responses [23]. Cytokines such as interferon (IFN)-γ, tumor necrosis factor (TNF)-α, interleukin (IL)−1, and IL-15 may induce CYP27B1 activity, thereby coupling inflammatory signaling with local vitamin D activation [23].

Vitamin D metabolism is tightly regulated by calcium, phosphate, parathyroid hormone (PTH), and fibroblast growth factor 23 (FGF23). High levels of 1,25(OH)_2_D and FGF23 induce 24-hydroxylase (CYP24A1), which inactivates vitamin D metabolites, maintaining homeostasis and preventing toxicity [23]. Pathological states such as chronic kidney disease (CKD), nephrotic syndrome, and malabsorptive disorders can disrupt these pathways, leading to deficiency [3].

The biological actions of vitamin D are mediated primarily through the vitamin D receptor (VDR), a nuclear receptor expressed in most human tissues. Upon ligand binding, the VDR heterodimerizes with the retinoid X receptor (RXR) and binds to vitamin D response elements (VDREs), modulating transcription of target genes [24]. GWAS have identified thousands of VDR binding sites across the human genome, many located near loci associated with autoimmune and neoplastic diseases [25]. VDR-mediated transcription is further refined by co-regulatory proteins and epigenetic modifications, contributing to tissue-specific responses [24]. In addition to classical genomic actions, vitamin D exerts rapid non-genomic effects via membrane-associated VDR and cytosolic signaling pathways. These non-genomic actions influence calcium flux, kinase activation, and cellular differentiation, and are mediated in part by the protein disulfide isomerase family member PDIA3 [26, 27].

The canonical physiological role of vitamin D is the regulation of intestinal calcium and phosphate absorption, which is essential for skeletal mineralization and bone integrity. Deficiency disrupts these processes, resulting in rickets, osteomalacia, and increased fracture risk [28]. Beyond skeletal health, vitamin D modulates innate and adaptive immunity, cell proliferation and apoptosis, cardiovascular and metabolic pathways, and neuroendocrine signaling, as supported by extensive experimental evidence [29]. A key immunological mechanism involves VDR-dependent induction of antimicrobial peptides, including cathelicidin (LL-37) and β-defensins, enhancing host defense. At the endocrine level, calcitriol suppresses renin gene transcription, providing a mechanistic link to blood pressure regulation and cardiovascular function. Genetic polymorphisms in VDR and vitamin D–metabolizing enzymes contribute to interindividual variability in disease susceptibility and response to supplementation [24].

Overall, vitamin D metabolism involves sequential hydroxylations in hepatic, renal, and extra-renal tissues, regulated by endocrine and paracrine factors. Its actions are predominantly genomic but complemented by rapid non-genomic signaling, underpinning both skeletal and extra-skeletal effects. These mechanisms provide the biological foundation for the context-dependent clinical effects of vitamin D observed across populations.

## Vitamin D and Health Outcomes

Circulating 25(OH)D has been extensively investigated in relation to a broad range of health outcomes. Although low 25(OH)D concentrations are consistently associated with increased morbidity and mortality, interpretation of these associations is complicated by confounding related to seasonality, geographic and genetic factors, adiposity, sun exposure, assay variability, and baseline vitamin D sufficiency, as well as by widespread supplement use. The rapid expansion of vitamin D research has therefore generated both enthusiasm and controversy, as many observational associations have not been confirmed in RCTs or MRS, suggesting that some relationships reflect underlying health status rather than direct causality.

To reconcile these discrepancies, this review applies a triangulated evidence model integrating mechanistic, genetic, observational, and interventional data, enabling discrimination between outcomes where vitamin D deficiency represents a modifiable causal risk factor and those in which associations are non-causal or context-dependent. Tables 1 and 2 summarize key evidence from MRS, meta-analyses, and RCTs across skeletal and extra-skeletal outcomes.

**Table 1:** List of some recent and major meta-analyses on circulating 25(OH)D levels in relation to skeletal and extra-skeletal outcomes

Meta-analyses (author, year)/main outcome	Studies	Participants	Key findings
Meta-analyses of MRS			
Fang A et al., 2024 [30] (Multiple health outcomes)	Umbrella meta-analysis of 133 MRS assessing 275 health outcomes across autoimmune, cardiovascular, skeletal, metabolic, neoplastic, neuropsychiatric and infectious disorders	> 1.4 million participants (from GWAS consortia and UK Biobank datasets)	-↑ genetically predisposed 25(OH)D concentrations were associated with: -strongly ↓ risk of multiple sclerosis (incidence and relapse) -weakly ↓ risk or dependent on broader genetic instruments (non-infectious uveitis and scleritis, psoriasis, femur and leg fracture, ALS, anorexia nervosa, delirium, heart failure, ovarian cancer, NAFLD, dyslipidemia and bacterial pneumonia) -↑ risk (Behçet’s disease, Graves’ disease, KSD, fracture of radium/ulna, BCC and overall cataracts) - Weak associations for most outcomes -Strongest, most consistent causal link with multiple sclerosis -Evidence for other outcomes remains suggestive.
Meng X et al., 2019 [31] (920 disease outcomes and 9 phenotypes)	-Single MR-PheWAS in the UK Biobank assessing 920 disease phenotypes -Background systematic review identified 63 prior MRS (not pooled).	339,256 participants	-No causal associations detected. -MRS showed null effects for SBP, DBP, hypertension, T2DM, IHD, BMI, depression, non-vertebral fracture, and all-cause mortality
Walker KC et al., 2025 [32] (congenital heart disease)	MRS across 3 pregnancy cohorts: ALSPAC-UK BiB- UK MoBa-Norway .	MVR: *N* = 8,722 pregnancies (77 CHD cases). MR: *N* = 74,953 pregnancies (646 CHD cases) Pregnancy 25(OH)D measured at 18–29w gestation;	No causal association was observed between maternal 25(OH)D concentrations during pregnancy and offspring CHD (OR 0.99 per SD increase; 95% CI 0.91–1.07).
Fusaro M et al., 2025 [33] (stroke)	MRS combined with large population-based cohorts (UK Biobank, EPIC-CVD, Copenhagen cohorts)	> 500,000 participants	-Non-linear association -Supplementation within the 50–70 nmol/L range was associated with a 13% ↓ stroke risk (pooled OR 0.87, 95% CI 0.76–1.00, *p* = 0.04). -No protective effect was found for vitamin D levels below 50 nmol/L or above 70 nmol/L. - Findings support a protective threshold window for optimal vitamin D status, suggesting targeted supplementation may reduce stroke risk
Zheng JS et al., 2020 [34] (type 2 diabetes)	EPIC-InterAct study EPIC-Norfolk study EPIC-CVD study Ely study SUNLIGHT consortium	Participants of European descent *N* = 120,618 for total 25(OH)D *N* = 40,562 for 25(OH)D3 *N* = 40,562 for C3-epi-25(OH)D3	-Observational analysis: Each ↑ 1-SD higher measured 25(OH)D level was associated with a 20% lower risk of T2DM (RR 0.80; 95% CI 0.77–0.84; *p* < 0.001). -MR analysis: A genetically predicted ↑ 1-SD 25(OH)D level showed no significant association with T2DM risk (OR 0.96; 95% CI 0.89–1.03; *p* = 0.23). -No causal relationship was found between total 25(OH)D or its metabolites and T2DM.
Najjar L et al., 2021 [35] (type 1 diabetes)	10 studies (7 case-control and 3 cohort) assessing associations with 7 vitamin D–related SNPs (*CYP2R1* rs10741657, *CYP2R1* rs117913124, *DHCR7/NADSYN1* rs12785878, *GC* rs3755967, *CYP24A1* rs17216707, *AMDHD1* rs10745742, *SEC23A* rs8018720) and *T1D* susceptibility.	> 120,000 participants (including UK Biobank and FinnGen datasets) -primarily of European ancestry.	-No significant associations between genetically determined 25(OH)D–related variants and T1DM risk (pooled OR 0.97–1.02; all *p* > 0.01). -Subgroup analysis in Caucasians confirmed null results.
Chen LJ et al., 2024 [36] (all-cause dementia, Alzheimer’s disease, and vascular dementia)	Single large prospective cohort analysis (UK Biobank)	269,229 adults aged 55–69 years (52% female). During follow-up: 7, 087 dementia cases (3,616 AD, 1,815 VD). 5.0% used vitamin D, 19.8% multivitamins.	-Vitamin D deficiency (< 30 nmol/L) associated with ↑ risk of all-cause dementia (HR 1.25; 95% CI 1.16–1.34), AD (HR 1.19; 95% CI 1.07–1.31), and VD (HR 1.24; 95% CI 1.08–1.43). -Insufficiency (30–50 nmol/L) associated with modestly ↑ risk (HR 1.10–1.15). -Supplement use linked to ↓ AD risk by 17% (HR 0.83; 95% CI 0.71–0.98) and ↓ VD risk by 14% (HR 0.86; 95% CI 0.75–0.98). -Protective associations strongest in participants aged 55–64 years and with BMI ≥ 30 kg/m²; absent in darker-skinned individuals. -↓ serum 25(OH)D predicts dementia risk, and supplementation may help prevent AD and VD.
Meta-analyses of observational studies and RCTs (+/- MRS)			
Liu D et al., 2022 [37] (overall health outcomes)	296 meta-analyses of observational studies (111 unique outcomes), 139 meta-analyses of RCTs (46 unique outcomes), and 73 Mendelian randomization studies (43 unique outcomes).	Aggregate evidence from > 280 publications including several million participants worldwide	-Across study types, ↓ 25(OH)D was causally associated with ↑ risk of all-cause mortality, AD, hypertension, schizophrenia, and T2DM. - Vitamin D supplementation showed a significant ↓ in all-cause mortality but no consistent benefit for specific diseases. For most other outcomes, observational associations were not confirmed by MR or RCTs. -Vitamin D deficiency appears causal for some chronic diseases, and supplementation may reduce overall mortality, but its disease-specific effects remain uncertain.
Theodoratou E et al., 2014 [38] (overall health outcomes)	−107 systematic literature reviews and 74 meta-analyses of observational studies of plasma vitamin D concentrations and 87 meta-analyses of RCTs with vitamin D supplementation - Covered 137 different health outcomes (skeletal, malignant, cardiovascular, autoimmune, infectious, metabolic)	->1 million participants across a wide range of populations and clinical settings. -Observational analyses: 5,000–80,000 participants per outcome - RCTs: 25,000 participants per meta-analysis.	- Observational links between higher 25(OH)D and many outcomes were not confirmed by RCTs. -No highly convincing causal evidence for vitamin D in any outcome. -Probable associations were found with ↑ birth weight, ↓ dental caries in children, ↑ maternal vitamin D at term, and ↓ PTH in dialysis patients. -Suggestive links included ↓ risks of colorectal cancer, hypertension, stroke, CVD, gestational diabetes, and T2DM, but results were not confirmed in RCTs. -No consistent benefit of vitamin D–only supplementation for bone density, fracture, or fall prevention. -Overall, vitamin D status appears more a marker of health than a causal factor.
Yao P et al., 2019 [39] (prevention of fractures)	11 observational studies (*n* = 39,141; 6,278 total fractures, 2,367 hip fractures) and 17 RCTs: 11 of vitamin D alone (*n* = 34,243) and 6 of calcium + vitamin D (*n* = 49,282).	-Observational cohorts: mean age ≈ 69 y; -RCTs included community-dwelling and institutionalized adults aged > 60 y, receiving 400–30,000 IU/d vitamin D (alone or + calcium 1000–1200 mg).	-Observational evidence: each 10 ng/mL (25 nmol/L) increase in 25(OH)D → 7% lower risk of any fracture (RR 0.93, 95% CI 0.89–0.96) and 20% lower hip-fracture risk (RR 0.80, 95% CI 0.75–0.86). -RCTs of vitamin D alone: no fracture protection (RR 1.06 for any fracture; RR 1.14 for hip). -RCTs of calcium + vitamin D: modest benefit—6% lower total fracture risk (RR 0.94, 95% CI 0.89–0.99) and 16% lower hip-fracture risk (RR 0.84, 95% CI 0.72–0.97). - Vitamin D alone, even at standard doses, does not prevent fractures; daily combined calcium + vitamin D supplementation shows modest efficacy, particularly in elderly or institutionalized populations.
Chakhtoura M et al., 2022 [40] (fractures in adults)	32 systematic reviews/meta-analyses	> 100,000 adults from community and institutional settings across RCTs	-Calcium + vitamin D reduced hip fracture risk (RR 0.61–0.84) and any fracture risk (RR 0.74–0.95), mainly in institutionalized older adults; no effect in community-dwelling individuals. Vitamin D alone showed no benefit (RR ≈ 1.0). -Combined Ca/D supplementation provides modest protection against fractures. -Greatest benefit occurs in elderly or institutionalized populations with low baseline 25(OH)D.
Winzenberg T et al., 2018 [41] (bone density in vitamin D-deficient children)	11 RCTs from 6 countries	1,439 healthy children/adolescents mean age: 10–18 y 86% female mean baseline 25(OH)D = 36 nmol/L; 79% <50 nmol/L	-Small benefit only for total hip BMD (Δ = +6.8 mg/cm²; 95% CI 0.7–12.9) -No effect on total body BMC, lumbar spine, femoral neck, or forearm. No clear interaction with baseline 25(OH)D (≥ or < 35/50 nmol/L). −1-year vitamin D supplementation yields no clinically meaningful improvement in BMD, even among vitamin D–deficient youth. -Routine supplementation in healthy children/adolescents is not supported
Dao D et al., 2015 [42] (stress fractures in military personnel)	9 observational studies (6 prospective cohort, 2 nested case-control, 1 case-control)	2,634 military personnel (age18-30 years; 44% male)	-serum 25(OH)D levels were significantly lower in SF cases than in controls. -No significant difference was seen in vitamin D levels between women with and without SF in prospective studies. -↓ in serum 25(OH)D levels during basic training in both SF cases and controls.
Mirhosseini N et al., 2018 [43] (CVD)	81 RCTs meeting strict inclusion criteria (≥ 3 months duration, pre/post 25(OH)D data, and improvement in vitamin D status).	9,993 participants (5,042 vitamin D; 4,951 placebo; mean age 55 ± 15 y; 67% female). Average dose ≈ 2,967 IU/d (range 400–12,000 IU/d); mean intervention ≈ 9.6 months	-Vitamin D supplementation (3,000 IU/day) ◊ significant ↑ in serum 25(OH)D levels, strong dose-response relationship -Vitamin D supplementation may protect from CVD by improving risk factors: -↓ in systolic and diastolic blood pressure (− 0.10 mmHg, *p* = 0.02, − 0.07 mmHg, *p* = 0.03, respectively), -↓ in triglycerides (− 0.12 mmol/L, *p* = 0.04), -↓ in total cholesterol (− 0.15 mmol/L, *p* = 0.009), -↓ in LDL cholesterol (− 0.10 mmol/L, *p* = 0.04). -↑ in HDL cholesterol (0.09 mmol/L, *p* = 0.05), -↓ in PTH levels (− 0.66 ng/L, *p* < 0.001), -↓ in hs-CRP levels (− 0.20 mg/L, *p* = 0.006) -No significant overall effect on pulse wave velocity - Benefits were strongest for doses ≥ 4,000 IU/d and achieved 25(OH)D ≥ 86 nmol/L, in vitamin D-deficient and/or obese participants. - Vitamin D improves blood pressure, lipids, and inflammatory biomarkers when adequate dosing and serum repletion are achieved
Huang J et al., 2017 [44] (myocardial infarction)	8 observational studies (7 case-control, 1 prospective cohort)	9,913 individuals, 3,411 MI patients and 6,502 non-MI controls	- MI patients had ↓ 25(OH)D than people without MI, especially in America and Asia -Sufficient vitamin D levels◊ ↓ in risk of heart attack (OR = 0.44, *p* = 0.004)
Nsengiyumva V et al., 2015 [45] (peripheral artery disease)	6 case-control studies	6,418 individuals, PAD patients: 1,217 Non-PAD controls: 5,201	-↓ 25(OH)D in PAD patients than controls -No difference in 25(OH)D levels in intermittent claudication subgroup - markedly ↓ 25(OH)D in critical limb ischemia subgroup vs. controls
Zhao R et al., 2022 [46] (pregnancy outcomes: gestational diabetes, pre-eclampsia)	69 prospective observational studies including cohort studies, case-cohort studies, or nested case-control studies	Pregnant women	-Higher maternal vitamin D levels are linked to ↓ risks of GDM, PE, and GH
Arayici ME et al., 2023 [47] (cancer risk)	35 meta-analyses of RCTs and observational studies assessing vitamin D intake and serum 25(OH)D in relation to cancer incidence and mortality. Included site-specific cancers: colorectal, breast, lung, prostate, liver, gastric, pancreatic, ovarian, and bladder.	> 1.5 million participants across global cohorts and trials.	**-**Both ↑ vitamin D intake and ↑ serum 25(OH)D levels were associated: -↓ overall cancer risk and mortality (intake OR 0.93 [95% CI 0.90–0.96]; 25(OH)D OR 0.80 [95% CI 0.72–0.89]; mortality RR 0.89 [95% CI 0.86–0.93]). - No significant association with cancer risk remained (OR = 0.99, *p* = 0.32) when only RCT-based meta-analyses were pooled - ↓ incidence for colorectal (OR = 0.89) and lung cancer (OR = 0.88) with higher vitamin D intake. **-** Vitamin D sufficiency and intake correlate with ↓ cancer incidence and mortality, though causality remains unproven and cancer type–specific effects are important.
Hossain S et al., 2019 [48] (breast cancer)	19 case-control/nested case-control and 3 prospective cohort studies.	229,597 adult females of reproductive age (pre-, postmenopausal or both)	-Vitamin D deficiency was associated with a ↑ risk of breast cancer (pooled RR 1.91; 95% CI 1.51–2.41; *p* < 0.001). -↑ total vitamin D intake (RR 0.99 per + 100 IU/day; 95% CI 0.97–1.00; *p* = 0.022) and supplemental vitamin D use (RR 0.97; 95% CI 0.95–1.00; *p* = 0.026) were associated with ↓ breast cancer risk.
Shen Y et al., 2024 [49] (pancreatic cancer)	13 case–control studies and 3 cohort studies	529,917 subjects of > 50 y.o. at baseline from different countries.	-No overall association between high 25(OH)D levels and pancreatic cancer incidence -↑ 25(OH)D levels were linked to ↓ pancreatic cancer mortality overall
Garland CF et al., 2017 [50] (colorectal cancer)	15 nested case-control and cohort studies conducted in 14 countries (10 in the U.S., 2 in Finland, 1 in Japan, 1 in Australia, and 1 European consortium), with prospective serum 25(OH)D levels	≈ 12,000 colorectal cancer cases and > 140,000 controls.	-↑ serum 25(OH)D levels strongly associated with ↓ CC risk. Pooled OR (highest vs. lowest quantile) 0.67 (95% CI: 0.59–0.76) -Linear dose–response: each 10 ng/mL ↑ corresponded to a proportional ↓ in risk. -OR 0.4 (95% CI: 0.2–1.0) at 50 ng/mL compared with 5 ng/mL, suggesting ~ 60% ↓ risk at optimal vitamin D status. -No U-shaped or adverse effects observed. - Maintaining serum 25(OH)D ≥ 35–50 ng/mL may significantly reduce CC incidence
Jung S et al., 2023 [51] (ovarian cancer)	15 observational studies	5,634 cases for vitamin D intake and 975 cases for blood 25(OH)D	- No significant association between total vitamin D intake and ovarian cancer risk (pooled RR 0.92; 95% CI 0.74–1.14). - ↑ blood 25(OH)D levels were associated with a 37% lower ovarian cancer risk (pooled RR 0.63; 95% CI 0.42–0.93). - Inverse association was stronger in case–control than in prospective studies, and supported by dose–response analyses.
Sluyter JD et al., 2020 [52] (clinical cancer outcomes)	> 40 meta-analyses: 35 based on observational studies and 8 on RCTs	> 1 million participants across cohort and RCT data. RCTs ranged from 18,000 to 83,000 participants, with up to 6,500 cancer incidence events and > 1,500 cancer deaths across major trials (VITAL, VIDA, WHI).	-Observational studies: ↑circulating 25(OH)D associated with ↓ total cancer incidence (RR 0.86, 95% CI 0.73–1.02) and ↓ cancer mortality (RR 0.81, 95% CI 0.71–0.93) -strong inverse links for CC and breast cancer, and 2% ↓ mortality per 20 nmol/L 25(OH)D ↑. - RCTs: Vitamin D supplementation had no effect on cancer incidence (pooled RR ~ 1.00) but consistently ↓ total cancer mortality by 13–16% (RR range 0.84–0.87, 95% CI 0.74–0.96). -Vitamin D supplementation ↓ total cancer mortality but not incidence.
Zhu B et al., 2015 [53] (COPD)	18 studies (8 cohort, 5 case-control, and 5 RCTs)	> 3,400 COPD patients and > 7,000 controls; 5 RCTs included 296 placebo and 300 vitamin D–treated participants.	-Serum 25(OH)D: No significant difference between COPD and controls in cohort studies (SMD = 0.19; 95% CI − 0.13–0.51), but ↓ levels in case–control studies (SMD 0.96; 95% CI 0.48–1.45). - Deficiency rates: Similar between COPD and controls (RR 0.96; 95% CI 0.75–1.21). However, vitamin D deficiency was significantly ↑ with COPD severity, ↓ 25(OH)D in moderate/severe vs. mild COPD (RR 0.72; 95% CI 0.63–0.83). --RCTs: Vitamin D supplementation ↓ COPD exacerbations, improved inspiratory muscle strength and maximal oxygen uptake, and benefited patients with baseline 25(OH)D < 20 ng/mL; no major adverse events reported
Feng H et al., 2017 [54] (in utero asthma & respiratory infections)	16 birth cohort studies assessed maternal or cord blood 25(OH)D and offspring respiratory outcomes	Pregnant women and their offspring in birth cohorts. Asthma: 8,871 participants (1,494 asthma cases), Wheeze: 9,072 participants (2,277 cases), Respiratory Tract Infections: 8,359 participants (2,562 cases)	-Comparing highest vs. lowest 25(OH)D categories, pooled ORs were 0.84 for asthma, 0.77 for wheeze, and 0.85 for respiratory tract infections (none statistically significant overall). -Cord blood 25(OH)D showed a significant inverse association with childhood wheeze (OR 0.43; 95% CI 0.29–0.62; *p* < 0.001). -↑ in utero vitamin D exposure may reduce risks of asthma and wheeze in offspring, consistent with RCTs of maternal supplementation.
Martínez-Lapiscina EH et al., 2020 [55] (multiple sclerosis)	13 prospective cohort studies	3,498 patients with CIS or RRMS.	1) Each + 25 nmol/L ↑ in serum 25(OH)D associated with: −10% lower clinical relapse rate (RR 0.90; 95% CI 0.83–0.99). −31% fewer gadolinium-enhancing lesions (RR 0.69; 95% CI 0.60–0.79). −14% fewer new/enlarging T2 lesions (RR 0.86; 95% CI 0.77–0.95). −19% fewer new active MRI lesions (RR 0.81; 95% CI 0.74–0.89). 2) ↑ 25(OH)D linked to ↓ inflammatory activity and relapses, but association with disability progression remains uncertain.
Clasen JL et al., 2023 [56] (rheumatoid arthritis)	3 prospective cohort studies, 3 nested case–control studies, 1 case–control study with retrospective exposure data	− 15,604 adult participants − 1,049 incident RA cases	-No significant association between pre-diagnostic 25(OH)D concentration and risk of developing RA (pooled RR per + 25 nmol/L 0.96; 95% CrI 0.82–1.13). -No association observed in men (RR 1.02) or women (RR 0.94) - Low 25(OH)D not linked to ↑ RA risk
Li J et al., 2024 [57] (autoimmune liver disease)	25 case–control studies	−3,487 patients (1,673 patients with AILD and 1,814 controls) −548 AIH cases, 1,106 PBC cases, and 19 PSC cases	- ↓ 25(OH)D₃ levels in both AIH and PBC, but total 25(OH)D did not differ significantly from controls. - ↓ Vitamins A and E in both AIH and PBC/PSC. -↓ Vitamin C only in PBC, not AIH. - ↓ Vitamin B6/B9 and ↑ vitamin B12/Hcy in PBC. -↓ Vitamin K1 and 1,25(OH)₂D₃ in patients with PBC - Multiple vitamin deficiencies occur in AILD
Štefanić M et al., 2020 [58] (autoimmune thyroiditis)	26 case–control studies	−2,695 cases with HT −2,263 controls	-Serum 25(OH)D levels were significantly lower in HT vs. controls (*p* < 0.001). -Odds of vitamin D deficiency (≤ 20 ng/mL) were 3.2-fold higher in HT (OR 3.21; 95% CI 1.94–5.30; *p* < 0.001). -Association consistent across regions (Asia/Europe), age groups (pediatric/adult), and study quality levels. -Latitude, BMI, and national income moderated effect size; greater differences at lower latitudes and in moderate-income settings. -HT patients have markedly↓25(OH)D; findings robust across populations but influenced by geographic and socioeconomic factors.
Du Y et al., 2020 [59] (Alzheimer’s disease)	9 RCTs	2,345 participants	-No significant cognitive benefit of vitamin D supplementation vs. control across all major cognitive domains: Mini-Mental State Examination, verbal fluency, verbal memory, visual ability and attention. -Subgroup analyses by calcium co-supplementation, follow-up duration, and baseline 25(OH)D levels did not alter results. -Vitamin D supplementation does not prevent AD or improve cognition
Xu Y et al., 2025 [60] (Parkinson’s disease)	Cross-sectional analysis of NHANES 2007–2018 combined with two-sample MR using GWAS data	NHANES cohort: 30,796 adults, of whom 1.1% with PD (mean age 61.9 ± 15.5 years; 68.5% vitamin D–insufficient). Genetic analysis: GWAS data from 401,460 individuals of European ancestry for 25(OH)D and 37,700 PD cases with 1.4 million controls.	-Observational results: No significant association between serum 25(OH)D and PD risk (OR ~ 0.7; 95% CI 0.4–1.4). -MRS: No causal link between Genetically predicted 25(OH)D and PD risk (OR 1.08; 95% CI 0.90–1.30; *P* = 0.39). Sensitivity and pleiotropy tests confirmed robustness. -Neither serum nor genetically determined 25(OH)D levels influence PD risk, suggesting that vitamin D deficiency is a non-causal biomarker rather than a risk factor.
Xu J et al., 2025 [61] (Parkinson’s disease)	8 RCTs across USA, Japan, Iran, Italy, and Poland.	646 patients with PD mean age ≈ 63.6 years; interventions included vitamin D alone or combined with calcium, whey protein, or probiotics. Doses ranged 1,000–10,000 IU/day, with follow-up 3–18 months.	-Vitamin D supplementation did not significantly improve motor scores, 10/8 m walk, or TUG test. -Vitamin D significantly increased 6-Minute Walking Distance by ~ 25 m. -Benefits were ↑ in younger patients and with doses ≥ 4,000 IU/day. -Vitamin D may modestly enhance walking endurance and muscle strength, supporting partial motor improvement but not global motor function in PD.
Musazadeh V et al., 2023 [62] (depression)	14 meta-analyses (10 RCTs and 4 cohort studies) and 3 cross-sectional analyses	RCTs included 24,510 participants; observational datasets encompassed ~ 104,600 individuals. Vitamin D doses ranged 2,500–6,000 IU/day, with interventions lasting 8–74 weeks.	-Pooled results from 10 RCT meta-analyses: Vitamin D supplementation significantly ↓ depression symptoms (SMD − 0.40; 95% CI − 0.60, − 0.21; *p* < 0.01). Stronger effects were observed for 4,000–5,000 IU/day doses and ≤ 20w duration. -Observational meta-analyses: Individuals with ↓ serum 25(OH)D had 1.6-fold higher odds of depression (OR1.60; 95% CI 1.08–2.36). -↑ 25(OH)D levels and supplementation (≥ 4,000 IU/day) are associated with significantly ↓ depressive symptoms and ↓ depression risk, especially in younger adults (≤ 50 y).
Schöttker B et al., 2014 [63] (mortality)	pooled data from 7 CHANCES cohorts (16 European countries) and NHANES III (USA).	−26,018 adults aged 50–79 years from Europe and the USA excluded: current smokers − 6,695 deaths occurred during follow-up (2,624 cardiovascular; 2,227 cancer).	-Lowest vs. highest 25(OH)D quintile associated with 57% higher all-cause mortality (RR 1.57; 95% CI 1.36–1.81). -Similar ↑ risk for cardiovascular mortality; cancer mortality association significant only in participants with a history of cancer (RR 1.70; 95% CI 1.00–2.88). -Relationship was inverse and curvilinear, consistent across age, sex, season, and country. - ↓ serum 25(OH)D linked to increased all-cause and cardiovascular mortality, independent of demographic and geographic factors
Zhu A et al., 2022 [64] (mortality with free vitamin D)	12 studies 10 on VDBP, 8 on bioavailable 25(OH)D, and 8 on free 25(OH)D).	~ 9,647 participants (range 148–5,899 per cohort; follow-up up to 20 years). Included European, Chinese, and U.S. populations.	1) Highest vs. lowest levels of: -Bioavailable 25(OH)D: 37% lower all-cause mortality (HR 0.63; 95% CI 0.46–0.87). -Free 25(OH)D: 29% ↓ all-cause mortality (HR 0.71; 95% CI 0.53–0.97). -Total 25(OH)D: 33%↓ all-cause mortality (HR 0.67; 95% CI 0.56–0.80). 2) ↑ VDBP associated with ↓ mortality only in cancer cohorts, not in the general population. 3) Bioavailable and free 25(OH)D show inverse associations with mortality similar to total 25(OH)D, offering no clear additional predictive value.
Zhang Y et al., 2018 [65] (mortality in dialysis patients)	18 cohort studies	14,154 adult maintenance dialysis patients.	1) Each 10 ng/mL ↑ serum 25(OH)D associated with: −22% lower all-cause mortality (RR 0.78; 95% CI 0.71–0.86). −29% lower cardiovascular mortality (RR 0.71; 95% CI 0.63–0.79). 2) Heterogeneity partly explained by differences in CVD prevalence, PTH levels, dialysis duration, and vitamin D assay methods. 3) ↑ serum 25(OH)D concentrations are significantly associated with ↓ all-cause and cardiovascular mortality among dialysis patients.

↓ decrease; ↑ increase; 25(OH)D: 25-hydroxyvitamin D; 25(OH)D₃: 25-hydroxyvitamin D₃; 1,25(OH)₂D₃: 1,25-dihydroxyvitamin D₃; AD: Alzheimer’s disease; AILD: Autoimmune Liver Disease; AIH: Autoimmune Hepatitis; ALS: Amyotrophic Lateral Sclerosis; ALSPAC: Avon Longitudinal Study of Parents and Children; AMSTAR-2: A Measurement Tool to Assess Systematic Reviews; BD: Behçet Disease; BCC: Basal Cell Carcinoma; BiB: Born in Bradford; BMC: Bone Mineral Content; BMD: Bone mineral density; BMI: Body Mass Index; Ca: Calcium; CHANCES: Consortium on Health and Ageing: Network of Cohorts in Europe and the United States; CC: Colorectal Cancer; CHD: Congenital Heart Disease; CI: Confidence Interval; CIS: Clinically Isolated Syndrome; CKD: Chronic Kidney Disease; COPD: Chronic Obstructive Pulmonary Disease; CrI: Credible Interval; CVD: Cardiovascular Disease; DBP: Vitamin D–Binding Protein; EPIC: European Prospective Investigation into Cancer and Nutrition; EPIC-CVD: European Prospective Investigation into Cancer and Nutrition–Cardiovascular Disease; EPIC-InterAct: European Prospective Investigation into Cancer and Nutrition–InterAct study; EPIC-Norfolk: European Prospective Investigation into Cancer and Nutrition–Norfolk study; FGF23: Fibroblast Growth Factor 23; GDM: Gestational Diabetes Mellitus; GH: Gestational Hypertension; GRS: Genetic Risk Score; Hcy: Homocysteine; HDL: High-Density Lipoprotein; HR: Hazard Ratio; HT: Hashimoto’s Thyroiditis; IHD: Ischemic Heart Disease; IU: International Units; IVW MR: Inverse-Variance Weighted Mendelian Randomization; KSD: Kidney Stone Disease; LDL: Low-Density Lipoprotein; MI: Myocardial Infarction; MoBa: Norwegian Mother, Father and Child Cohort Study; MR: Mendelian Randomization; MR-PheWAS: Mendelian Randomization – Phenome-Wide Association Study; MRI: Magnetic Resonance Imaging; MS: Multiple Sclerosis; MVR: Multivariable regression; NAFLD: Nonalcoholic Fatty Liver Disease; NHANES: National Health and Nutrition Examination Survey; OR: Odds Ratio; PAD: Peripheral Artery Disease; PBC: Primary Biliary Cholangitis; PD: Parkinson’s disease; PE: Preeclampsia; PTH: Parathyroid Hormone; PRS: Polygenic Risk Score; PSC: Primary Sclerosing Cholangitis; RCT: Randomized Controlled Trial; RR: Relative Risk; RRMS: Relapsing-Remitting Multiple Sclerosis; SBP: Systolic Blood Pressure; SD: Standard Deviation; SF: Stress Fracture; SMD: Standard Mean Difference; SNP: Single-Nucleotide Polymorphism; SUNLIGHT: Study of Underlying Genetic Determinants of Vitamin D and Highly Related Traits; T1DM: Type 1 Diabetes Mellitus; T2DM: Type 2 Diabetes Mellitus; TUG: Timed Up and Go test; VDBP: Vitamin D–Binding Protein; VD: Vascular Dementia; VDR: Vitamin D Receptor; Vit-D: Vitamin D; VITAL: Vitamin D and Omega-3 Trial

**Table 2:** List of recent and important RCTs on vitamin D supplementation and skeletal and extra-skeletal outcomes

RCT/study	Country	*N*	Participant characteristics	Dose and Frequency	Duration	Key findings with vitD supplementation compared to placebo (+ reference)
VITAL (VITamin D and OmegA-3 Trial) Randomized double-blind, placebo-controlled, 2 × 2 factorial design [66–71]	USA	25,871	-mean age: 67.1 ± 7.1 y -men ≥ 50 y, women ≥ 55 y − 51% female - ~72% non-Hispanic white -BMI: 28.1 ± 5.7 kg/m² -mean baseline 25(OH)D: 30.8 ± 10 ng/mL -no prior CVD or cancer	Vitamin D3 2,000 IU/day +/− marine omega-3 1 g/day	~ 5.3 y	-Primary endpoints (**cancer & CVD**): No significant reduction in major CVD events or invasive cancer incidence. -No effect on MACE -**Cancer mortality/advanced cancer**: 17% lower risk of metastatic or fatal cancer, mainly among non-obese participants. -**Fracture outcomes** (skeletal): No reduction of total, non-vertebral or hip fractures. Participants were typically in good overall health and were not specifically chosen based on vit D deficiency, reduced bone density, or a diagnosis of osteoporosis. - **Falls** (skeletal/neuromuscular): No reduction in ≥ 2 falls that required medical attention or hospitalization or injurious falls - **T2DM**: No reduction in incident T2DM overall; possible benefit at ≥ 40 ng/mL 25(OH)D. - **Autoimmune disease**: 22% ↓ risk of autoimmune disease with vitamin D supplementation.
ViDA [Vitamin D Assessment (ViDA) study] Randomized double-blind, placebo-controlled trial [72, 73]	New Zealand	5,108	-mean age: 65.9 ± 8.3 y − 58% male ~83% European - mean BMI: 28.5 ± 5.1 kg/m² - baseline 25(OH)D: ~63 ± 24 nmol/L (vitamin D–replete).	200,000 IU vitamin D₃ loading dose + 100,000 IU/month thereafter.	3.3 y	-**Primary endpoint (CVD)**: No benefit on major CVD events (HR 1.02; 95% CI 0.87–1.20). -In one sub-study, modest ↓ central systolic BP among vitamin D–deficient participants. -**Cancer**: No effect on cancer incidence and death -**Mortality**: No effect on all-cause mortality -**Skeletal outcomes & Falls**: No benefit on total fractures or falls (HR ≈ 1.0). -Monthly high-dose vitamin D did not improve skeletal or extra-skeletal outcomes in generally healthy, vitamin D–replete adults.
D-Health [Vitamin D for Health Trial] Randomized double-blind, placebo-controlled population trial [74, 75]	Australia	21,315	-mean age: 69.3 ± 5.5 y −49.6% female -mean BMI ≈ 28 kg/m² ≈ 96% White -baseline 25(OH)D ≈ 77 ± 25 nmol/L (placebo) vs. 115 ± 30 nmol/L (vitamin D).	Vitamin D₃ 60 000 IU monthly.	5.7 y (median)	- **Primary endpoints: all-cause mortality and cancer**: 1) No reduction in all-cause mortality or total cancer incidence. 2) No reduction in cancer mortality. 3) In exploratory analyses, ↑ cancer mortality for those who receive vitamin D, when the first 2 years of follow-up were excluded from analysis. -**CVD**: No overall cardiovascular benefit; exploratory analyses showed a non-significant ~ 19% lower MI risk. -**Skeletal outcomes**: No reduction in total fractures, falls or BMD loss. Possible ↑ fracture risk in BMI < 25 kg/m² subgroup -Monthly high-dose vitamin D did not improve skeletal or extra-skeletal outcomes in vitamin D–replete older adults.
DO-HEALTH [Vitamin D, Omega-3, and Home Exercise] Multicenter, double-blind, placebo-controlled, 2 × 2 × 2 factorial RCT [76–78]	Europe: 7 European cities (Zürich, Basel, Geneva, Toulouse, Berlin, Innsbruck and Coimbra).	2,157	-mean age: 74.9 ± 4.4 y -−61.7% female -mean BMI: 26.5 ± 4.4 kg/m² - Participants of European ancestry ~ 41% with 25(OH)D < 20 ng/mL (mean ≈ 22 ng/mL).	Vitamin D₃ 2,000 IU/day + Omega-3 1 g/day + SHEP (30 min, 3× per week).	3 y	**Primary endpoint**: a composite of six outcomes (fracture, infection, cancer, CVD, cognitive decline, or death), representing healthy ageing -No benefit on ↓ nonvertebral fractures and total falls -No improvement in SBP or DBP -No benefit on MACE prevention -No change in lipidemic biomarkers (TG, CHO, HDL-C, LDL-C, non-HDL) - No differences in infection rates or cognition - Combined vitamin D + omega-3 + exercise associated with ~ 39% ↓ cumulative cancer risk in healthy, active adults aged ≥ 70 years, who were largely vitamin D–replete -Vitamin D₃ 2 000 IU/day alone provided no measurable skeletal or cardiovascular benefit, while combination therapy modestly reduced cancer risk in active, largely vitamin D-replete older adults.
FIND (Finnish Vitamin D Trial) Randomized, double-blind, placebo-controlled, parallel-group RCT [79, 80]	Finland	2,495	-mean age: 68.2 ± 5.5 −42.8% female - mean BMI: 27.1 ± 4.3 kg/m² −100% White -baseline 25(OH)D: 29.9 ± 7.3 ng/mL (vitamin D–replete).	Vitamin D₃ 1,600 IU/day or 3,200 IU/day vs. placebo.	4.3 y Median 5y	-**Primary outcomes**: No effect on major CVD events or invasive cancer incidence **-**No difference in all-cause or cause-specific mortality -No reduction in incident T2DM, though possible benefit in participants with baseline 25(OH)D < 50 nmol/L -High-dose daily vitamin D₃ (1,600–3,200 IU) did not reduce CVD, cancer, T2DM, or mortality in vitamin D–replete older adults, though benefit may exist for deficient individuals.
D2d (Vitamin D and Type 2 Diabetes) Multicenter, randomized, double-blind, placebo-controlled trial [81–83]	USA	2,423 prediabetic adults	-mean age: 60.0 ± 9.9 y − 45% female -mean BMI: 32.1 ± 4.5 kg/m² − 66.7% White - mean baseline 25(OH)D: 28 ± 10.2 ng/mL	Vitamin D₃ 4 000 IU/day vs. placebo.	2.5 y (median)	-**Primary outcome**: No significant reduction in T2DM incidence (HR 0.88; 95% CI 0.75–1.04; *p* = 0.12). -↓ T2DM risk among participants attaining 25(OH)D ≥ 40 ng/mL (exploratory). -No effect on total cancer incidence or colorectal adenomas/serrated lesions -Vitamin D₃ 4 000 IU/day did not prevent T2DM overall, though higher achieved 25(OH)D levels may confer benefit; no effect on cancer or colorectal polyp risk.

↓ decrease; ↑ increase; BMD: bone mineral density; BMI: body mass index; BP: blood pressure; CHO: cholesterol; CI: confidence interval; CVD: cardiovascular disease; DBP: diastolic blood pressure; HDL-C: high-density lipoprotein cholesterol; HR: hazard ratio; IU: international units; LDL-C: low-density lipoprotein cholesterol; MACE: major adverse cardiovascular events (coronary heart event or intervention, heart failure, stroke); MI: myocardial infarction; non-HDL: non-high-density lipoprotein; SBP: systolic blood pressure; SHEP: simple home strength exercise program; TG: triglycerides; T2DM: type 2 diabetes mellitus; y: years

### Vitamin D and Skeletal Outcomes

Vitamin D plays a central role in bone metabolism by promoting intestinal absorption of calcium and phosphate, facilitating mineralization, and maintaining serum calcium homeostasis. Deficiency impairs these processes, leading to secondary hyperparathyroidism, increased bone turnover, and risk of osteoporosis, osteomalacia and fractures [28]. Vitamin D deficiency is the primary cause of rickets and osteomalacia, and vitamin D supplementation is the established first-line treatment and preventive strategy for both conditions. Triangulated evidence from observational studies, MRS, RCTs, and meta-analyses strongly supports vitamin D supplementation as both preventive and therapeutic for rickets and osteomalacia, with added benefit from calcium in deficient populations [26, 84–86]. Moreover, monogenic defects in *CYP27B1* (VDDR type 1 A), *CYP2R1* (VDDR type 1B), or *VDR* (VDDR type 2 A/2B) cause rachitic phenotypes that resolve with appropriate vitamin D (or active analog) therapy, thereby providing compelling evidence of causality that impaired vitamin D synthesis/signaling leads to rickets [87, 88]. Notably, maternal vitamin D supplementation during pregnancy and lactation may reduce the risk of infantile biochemical rickets in populations without routine infant supplementation [89].

Based on epidemiological data, an estimate of 5% to 10% of hip fractures is attributable to vitamin D deficiency, particularly in older adults [90]. Meta-analyses of observational studies consistently have shown that low 25(OH)D levels are associated with an increased risk of fractures across age groups and populations, higher occurrence of stress fractures, falls, frailty, and lower bone mineral density (BMD) [42, 91, 92]. Individuals in the lowest 25(OH)D categories have a significantly higher risk of hip fracture compared to those in the highest categories, with adjusted RRs ranging from 1.4 to 1.8 in elderly populations [91, 93]. Each 25 nmol/L (10 ng/mL) increase in 25(OH)D is associated with a 7% lower risk of any fracture and a 20% lower risk of hip fracture [39]. The risk is most pronounced at serum 25(OH)D levels below 50–60 nmol/L, and is observed across both men and women [94–96]. In pediatric populations, serum 25(OH)D concentrations ≤ 50 nmol/L are associated with increased fracture risk (pooled OR 1.29, 95% CI 1.10–1.53) [92].

In contrast, MRS have shown that genetically low 25(OH)D is not causally associated with an increased risk of fractures or reduced BMD in the general population [30, 31, 97]. Recent large RCTs, including VITAL, ViDA, D-Health and DO-HEALTH, consistently showed no benefit of vitamin D supplementation on fractures or falls among generally healthy, vitamin D–replete older adults [66, 68, 72, 74, 76]. The absence of skeletal benefits in these trials likely reflects adequate baseline 25(OH)D levels, heterogeneous dosing regimens, and limited contrast between intervention and control groups.

On the contrary, earlier trials have reported modest fracture reduction (~ 10%) predominantly in targeted frail or institutionalized populations with markedly low baseline 25(OH)D and frequently with co-administered calcium [98–100]. Umbrella reviews and meta-analyses of RCTs indicate that vitamin D supplementation alone does not significantly reduce fracture risk, falls, or improve BMD in community-dwelling adults [37, 38]. However, combined vitamin D and calcium supplementation is effective in reducing hip and total fracture risk, particularly in institutionalized or high-risk elderly populations [40]. Evidence for reductions in falls or improvements in frailty remains limited and inconsistent [38]. Combined calcium and vitamin D supplementation may enhance pelvic BMD and correct serum 25(OH)D insufficiency in postmenopausal women with osteoporosis, but without significantly reducing the risk of clinical fractures [101].

In children and adolescents, meta-analyses indicate only small increases in hip BMD after approximately one year of vitamin D supplementation, with no clinically meaningful effects at other skeletal sites [41]. Accordingly, routine vitamin D supplementation for fracture prevention in vitamin D–replete pediatric populations is not supported by current evidence.

In summary, vitamin D supplementation is clearly effective for the prevention and treatment of rickets and osteomalacia but has no convincing causal effect on BMD or fracture prevention in vitamin D–replete adults. Modest fracture risk reductions are observed only with combined calcium and vitamin D supplementation in deficient or institutionalized elderly populations, while gains in bone density in children and adolescents are minimal.

### Vitamin D and Extra-Skeletal Outcomes

Beyond skeletal health, vitamin D has been extensively investigated in relation to a wide range of extra-skeletal outcomes, including cardiovascular, metabolic, neoplastic, autoimmune, infectious, and neuropsychiatric disorders. Although low circulating 25(OH)D concentrations are consistently associated with increased risk of multiple chronic diseases and mortality, causal interpretation remains challenging. Accordingly, the following sections synthesize triangulated evidence across major extra-skeletal outcomes, highlighting areas of convergence, inconsistency, and ongoing controversy.

#### Vitamin D and Cardiovascular Disease

Cardiovascular disease (CVD) remains the leading cause of morbidity and mortality worldwide, with prevalence and disease burden continuing to rise across both developed and developing regions. Hypertension affects over 1.2 billion individuals globally, while ischemic heart disease, heart failure (HF), atrial fibrillation (AF), peripheral arterial disease (PAD), and stroke account for the majority of cardiovascular morbidity and mortality [102]. These data underscore the public health relevance of investigating potentially modifiable exposures, including vitamin D status.

Mechanistic and experimental studies have proposed several cardioprotective actions of vitamin D. VDR expression in cardiomyocytes, vascular smooth muscle cells, and endothelial cells enables calcitriol to modulate vascular tone, inhibit cardiac hypertrophy and fibrosis, and regulate inflammation and oxidative stress [103]. Experimental models further demonstrate reduced foam cell formation, improved endothelial function, enhanced nitric oxide bioavailability, and reduced thrombogenicity, collectively supporting the anti-atherogenic effects of vitamin D [103]. However, translation of these findings into clinical benefit remains uncertain.

Evidence from MRS examining genetically determined 25(OH)D concentrations and cardiovascular outcomes has been largely null. Large-scale analyses have shown no causal associations between genetically predicted vitamin D levels and hypertension, CHD, MI, stroke, or all-cause mortality [31, 104]. Meta-analyses of MRS report weak or inconsistent associations for selected outcomes, such as HF or dyslipidemia, without robust evidence of causality [30]. By contrast, a recent MRS reported inverse associations for angina, CHD and lacunar stroke, but no causal effects for incident hypertension or MI [105]. Some recent analyses suggest a modest reduction in stroke risk (~ 13%) within a narrow 25(OH)D range (50–70 nmol/L; pooled OR 0.87, 95% CI 0.76–0.99), but overall, findings remain inconsistent and hypothesis-generating [33, 104].

Observational meta-analyses and umbrella reviews have reported inverse associations between low 25(OH)D levels and CVD outcomes, including PAD, but with considerable heterogeneity and risk of bias [44, 45]. The umbrella review by Theodoratou et al. found no convincing evidence for a causal role of vitamin D in CVD [38]. A more recent umbrella review integrating observational studies, MRS and RCTs confirmed that although low vitamin D concentrations are associated with increased CVD risk, the strength and consistency of these associations are modest [37]. Meta-analyses of cohort studies show inverse, non-linear relationships between 25(OH)D and CHD, stroke and mortality, with steeper risks at very low concentrations (< 30 nmol/L) and attenuation above ~ 50 nmol/L [104, 106]. Consequently, low vitamin D status may act primarily as a marker of adverse cardiovascular risk rather than a causal mediator.

Turning to RCTs, the evidence for cardiovascular benefit from vitamin D supplementation has been largely disappointing. A 2023 meta-analysis of more than 80 RCTs (~ 160,000 participants) reported a modest reduction in all-cause mortality (OR 0.95, 95% CI 0.91–0.99) but no significant effect on CV mortality, MI, stroke, HF, or MACEs [107]. A focused meta-analysis of 18 trials (70,278 participants) similarly found no effect on CVD mortality or total CV events [108]. A very recent meta-analysis reported no significant reduction in MACEs, stroke or CV death, with only a borderline reduction in MI risk (HR 0.88; 95% CI 0.77–1.01), with a benefit more pronounced in participants with BMI ≥ 25 kg/m² [109]. Large trials including VITAL, ViDA, D-Health, DO-HEALTH and FIND consistently showed no reduction in MACEs among generally healthy, vitamin D–replete adults [66, 72, 75, 76, 79]. Accordingly, current RCT evidence does not support routine vitamin D supplementation for primary prevention of cardiovascular disease.

In summary, despite biologically plausible mechanisms and consistent observational associations, triangulated evidence does not support a strong causal role for vitamin D in the prevention of CVD. Any potential benefit appears limited to specific subgroups or narrow exposure ranges and has not been consistently demonstrated across MRS or RCTs, reinforcing the need for cautious interpretation of observational findings.

#### Vitamin D and Cancer

Cancer remains a major global health burden and a leading cause of morbidity and mortality worldwide. According to recent estimates, approximately 20 million new cancer cases and nearly 10 million cancer-related deaths occur annually, with breast, lung, colorectal, prostate, and gastric cancers accounting for the highest incidence [110]. These figures highlight the continued interest in identifying modifiable factors, including nutritional and hormonal determinants such as vitamin D status.

Mechanistic and animal studies provide biologically plausible links between vitamin D signaling and carcinogenesis. Calcitriol regulates cell proliferation, differentiation, apoptosis, and angiogenesis through VDR–mediated transcriptional control and modulation of key oncogenic pathways, including Wnt/β-catenin, NF-κB, and growth factor signaling. In preclinical models, vitamin D or VDR agonists may reduce tumor growth and inhibit metastasis in a tissue- and dose-dependent manner, modulate the gut microbiome and enhance antitumor immunity [111–113]. However, mechanistic plausibility alone does not establish clinical causality.

Evidence from MRS has not demonstrated a causal association between genetically determined circulating 25(OH)D concentrations and overall cancer incidence or site-specific cancer risk, including colorectal, breast, lung or prostate cancers [30, 31, 114–116]. Similarly, large MR analyses do not support a causal role for vitamin D status in cancer mortality [30, 115]. The available evidence is robust in showing null associations, but the number of studies specifically addressing cancer mortality is relatively small, and precision for less common cancer types is limited.

In contrast, umbrella reviews and meta-analyses of observational studies have consistently reported inverse associations between low circulating 25(OH)D and cancer mortality, particularly for colorectal, breast, and lung cancers, whereas correlations with incidence are weaker and heterogeneous [47, 50, 52, 117, 118]. Higher vitamin D intake has been associated with a 13–16% reduction in total cancer mortality, although effects on overall cancer risk remain modest and inconsistent [47, 52]. These observational associations are susceptible to confounding and reverse causation, particularly in advanced or preclinical disease states [49, 51, 119].

With respect to RCTs, evidence is more conservative. Meta-analyses indicate that vitamin D supplementation does not reduce total cancer incidence in the general population, but is associated with a modest reduction in total cancer mortality (RR 0.88, 95% CI 0.80–0.96) [47, 52]. These mortality benefits appear consistent across large trials but remain small in magnitude, with subgroup analyses suggesting greater benefit among individuals with low baseline 25(OH)D or specific cancer subtypes [79]. No significant effect on site-specific cancer incidence has been demonstrated in RCTs [66, 73, 75, 79, 82]. The VITAL trial reported no reduction in cancer incidence but a lower risk of metastatic or fatal cancer, especially among non-obese participants [66], whereas intermittent high-dose regimens have generally failed to show any benefit [85].

In summary, triangulated evidence does not support a causal role for vitamin D in cancer prevention. Nevertheless, a modest reduction in cancer-specific mortality with vitamin D supplementation, especially with daily dosing regimens, is supported by RCT and meta-analytic data, particularly in deficient populations, suggesting potential benefit in disease progression or survival rather than cancer initiation.

#### Vitamin D and Metabolic Disorders

Metabolic disorders, including T2DM, T1DM, dyslipidemia, and metabolic dysfunction–associated steatotic liver disease (MASLD), represent major contributors to global morbidity and mortality. VDR and vitamin D–activating enzymes are expressed in pancreatic β-cells, hepatocytes, adipose tissue, and skeletal muscle, providing a biological basis for potential metabolic effects. Calcitriol enhances insulin secretion, improves insulin sensitivity, activates the AMPK signaling, and exerts anti-inflammatory effects through the suppression of NF-κB and pro-inflammatory cytokines such as IL-6 and TNF-α [120]. In rodent models, vitamin D deficiency worsens glucose intolerance, dyslipidemia, hepatic steatosis, and adipose inflammation while vitamin D sufficiency improves glycemia and hepatic lipid metabolism [120]. These findings provide biological plausibility but do not establish causality in humans.

Evidence from MRS does not support a causal role for genetically determined 25(OH)D concentrations in the development of T2DM, T1DM, gestational diabetes or dyslipidemia [30, 34, 35, 37, 121, 122]. For MASLD, genetic evidence is limited, with a recent bidirectional MRS involving more than 450,000 Europeans reporting a ~ 22% lower risk per 1-SD increase in genetically predicted 25(OH)D, without evidence of a reverse causal effect [123].

In contrast, epidemiologic syntheses have consistently associated lower 25(OH)D with higher T2D, gestational diabetes and T1DM occurrence, worse insulin resistance, adverse lipid profiles, and greater MASLD burden. Low 25(OH)D is also linked to poorer glycemic control, metabolic syndrome, and increased risk of diabetic complications [46, 124–128].

With respect to RCTs, the D2d trial (4,000 IU/day cholecalciferol) reported a non-significant 12% reduction in diabetes incidence [81]. A pooled analysis of D2d (USA), Tromsø (Norway), and DPVD (Japan) trials showed a 15% reduction in the progression to T2DM and a 30% higher likelihood of return to normoglycemia, despite individual trials missing statistical significance [129]. Individuals with prediabetes or marked vitamin D deficiency (< 30 nmol/L) appear to derive modest benefit, particularly when achieved serum 25(OH)D exceeds 100 nmol/L [100, 129]. Meta-analyses of RCTs in established T2DM demonstrate small improvements in insulin resistance, fasting glucose, HbA1c, lipid and inflammatory parameters, largely confined to vitamin D–deficient individuals [130, 131].

For dyslipidemia, umbrella reviews indicate small and inconsistent lipid effects, most evident for triglycerides and in deficient individuals [132]. For MASLD and T1DM, evidence for clinical benefit from vitamin D supplementation remains limited and inconclusive [124, 133–135]. RCT meta-analyses in MASLD have shown improved 25(OH)D levels without consistent benefits on liver enzymes, insulin resistance, or triglycerides, and minor LDL-C effects that are heterogeneous and not clinically significant [133, 134]. Definitive RCT evidence for vitamin D supplementation is lacking for T1DM prevention or sustained metabolic benefit in newly diagnosed disease [135].

Overall, despite biologically plausible mechanisms and consistent observational associations, current genetic and interventional evidence does not support a causal or universal therapeutic role for vitamin D supplementation in T2DM, dyslipidemia, MASLD, or T1DM. Potential benefits appear limited to deficient or high-risk subgroups, and vitamin D supplementation should be considered supportive rather than a primary metabolic therapy.

#### Vitamin D and Obesity

Overweight and obesity have become a global health crisis, currently affecting an estimated 2.11 billion adults, representing approximately 45% of the adult population. Projections indicate that overweight and obesity prevalence will continue to rise globally, with a growing burden in lower-income countries [136]. Obesity represents a major risk factor for T2DM, CVD, MASLD, cancer, and all-cause mortality, being consistently associated with lower serum 25(OH)D concentrations [12, 136]. Proposed mechanisms include volumetric dilution, sequestration of vitamin D in adipose tissue, reduced sun exposure, and altered vitamin D metabolism. Conversely, low vitamin D status has been hypothesized to contribute to adipogenesis, chronic low-grade inflammation, and insulin resistance, suggesting a potential bidirectional relationship [12].

Experimental studies indicate that vitamin D signaling may influence adipocyte differentiation, lipid metabolism, and inflammatory pathways. Calcitriol inhibits adipogenesis through the suppression of peroxisome proliferator–activated receptor-γ (PPAR-γ) and reduces adipose tissue macrophage infiltration and pro-inflammatory cytokine production (IL-6, TNF-α, MCP-1) in animal models [137]. Interestingly, maternal vitamin D sufficiency may also mitigate adverse adipose tissue programming in the offspring [138]. These mechanistic findings support biological plausibility but do not establish directionality in humans.

MRS have clarified the directionality of the vitamin D–obesity relationship. A large bidirectional MRS of 42,024 UK Biobank participants has shown a unidirectional relationship, whereby genetically higher BMI leads to lower circulating 25(OH)D, whereas genetically higher 25(OH)D has minimal effect on BMI [139]. However, subgroup analyses in severe deficiency (< 25 nmol/L) suggested small causal effects on visceral adiposity and metabolic syndrome traits.

Meta-analyses have found a strong inverse association between low 25(OH)D and obesity, with individuals with obesity having a 35% higher prevalence of vitamin D deficiency than normal-weight controls [37, 140]. Low 25(OH)D concentrations are also associated with higher inflammatory markers and adverse metabolic profiles in obesity [141, 142]. Prospective cohort studies show that lower baseline 25(OH)D predicts higher risk of metabolic syndrome and central obesity (RR 1.25; 95% CI 1.11–1.41), although reverse causation cannot be excluded [143].

RCTs and umbrella reviews indicate that vitamin D supplementation does not produce clinically meaningful reductions in body weight, BMI, waist circumference, waist-to-hip ratio or fat mass, although modest reductions in fat mass or inflammatory markers have been reported in deficient individuals [144–147]. Vitamin D combined with caloric restriction may modestly enhance fat loss [148]. An umbrella review of 31 RCTs in 3,856 prediabetic individuals found no significant effect on BMI of vitamin D supplementation (400 − 60,000 IU/week for 8–52 weeks) but reported improvements in glycemic indices and triglycerides, particularly with doses > 30,000 IU per week, administration duration longer than 24 weeks, younger age (< 50 years old), and baseline deficiency [149]. Large trials, including D2d and VITAL, reported no effect of vitamin D supplementation on body composition or incident diabetes [69, 81, 150].

In summary, triangulated evidence indicates that obesity is a determinant of low vitamin D status rather than a consequence of vitamin D deficiency. Vitamin D supplementation does not meaningfully reduce body weight or adiposity; however, modest improvements in metabolic or inflammatory profiles may occur in deficient individuals. The ES recommends against routine vitamin D screening or supplementation in otherwise healthy adults with obesity [3].

#### Vitamin D and Autoimmune Disorders

Autoimmune diseases represent a growing cause of chronic morbidity worldwide and affect approximately 4–10% of the global population, with marked variation by age, sex, and geographic region [151–154]. Vitamin D has attracted particular interest in this context because of its established immunomodulatory properties and widespread expression of the VDR in immune cells.

Mechanistic and animal studies have shown that calcitriol modulates both innate and adaptive immunity by promoting regulatory T-cell differentiation, regulating dendritic cell maturation, suppressing Th1/Th17 responses, and attenuating NF-κB–mediated inflammatory signaling. In experimental models of multiple sclerosis (MS), inflammatory bowel disease, and autoimmune thyroiditis, vitamin D or VDR agonists reduce disease severity and inflammatory infiltration [155, 156]. These mechanisms provide biological plausibility for a role of vitamin D in immune tolerance.

Evidence from MRS provides the strongest causal support among extra-skeletal outcomes. Genetically lower 25(OH)D concentrations are causally associated with an increased risk of MS, including both disease incidence and relapse activity [30, 157]. Suggestive genetic associations have also been reported for psoriasis, whereas findings for RA, systemic lupus erythematosus (SLE), and autoimmune thyroid disease are inconsistent or weak [30, 157, 158].

Meta-analytic syntheses of observational studies consistently report associations between lower circulating 25(OH)D and increased risk of several autoimmune diseases, particularly MS, SLE, and RA, although heterogeneity and residual confounding remain important limitations [37, 55, 56, 159, 160]. Randomized controlled trial evidence remains limited but informative. In the VITAL trial, vitamin D₃ supplementation (2,000 IU/day) reduced the incidence of newly diagnosed autoimmune diseases by approximately 22% compared with placebo. Benefits were most evident during the active supplementation period [161]. For established autoimmune diseases, RCTs have yielded heterogeneous results, with modest reductions in disease activity or relapse rates reported in selected conditions, such as MS and SLE, primarily among vitamin D–deficient populations, but without consistent disease-modifying effects [162–164].

Overall, vitamin D exhibits immunoregulatory properties that may confer protection in selected autoimmune disorders, with the clearest causal evidence observed in MS.

#### Vitamin D and Neuropsychiatric/Neurodegenerative Disorders

Neuropsychiatric and neurodegenerative disorders represent a major and growing global health burden [165]. Depression affects more than 300 million individuals worldwide, while over 55 million people live with dementia, with Alzheimer’s disease (AD) accounting for the majority of cases [165]. Parkinson’s disease (PD) and other neurodegenerative disorders are also increasing in prevalence, largely driven by population aging [165].

Vitamin D functions as a neurosteroid, with VDR and 1α-hydroxylase being expressed in neurons, astrocytes, and microglia in brain regions involved in cognition, mood regulation, and motor control, including the hippocampus, substantia nigra, and cortex [166]. Experimental studies suggest that vitamin D signaling influences neuroinflammation, oxidative stress, neurotransmission, mitochondrial function, and neurotrophin synthesis, including the brain-derived neurotrophic factor (BDNF) [166, 167]. These mechanisms provide biological plausibility for the role of vitamin D in brain health.

Observational studies and meta-analyses have consistently reported associations between low circulating 25(OH)D concentrations and increased risk of depression, cognitive decline, dementia, AD, PD and ALS [62, 168–172]. Lower 25(OH)D has also been associated with greater symptom severity and poorer functional outcomes in PD [170, 171]. Moreover, observational evidence links vitamin D deficiency to schizophrenia risk, supported by neonatal cohort data showing inverse associations between neonatal 25(OH)D and later schizophrenia [173–175].

However, MRS provide limited support for causality. Large-scale analyses show no consistent or weak causal association between genetically determined 25(OH)D concentrations and depression, PD, schizophrenia, ALS, fibromyalgia and chronic pain syndromes [60, 176–179]. Some MRS suggest possible non-linear or threshold effects for dementia risk at 25(OH)D concentrations below 25–30 nmol/L, but findings remain inconsistent [36, 180].

RCTs have largely failed to demonstrate clear neuropsychiatric benefits of vitamin D supplementation. Large trials and meta-analyses report no significant reduction in incident depression, cognitive decline, or dementia with vitamin D supplementation in generally vitamin D–replete populations [59, 181–184]. Small or heterogeneous improvements in depressive symptoms or selected motor outcomes in PD have been reported, primarily in deficient individuals or with short-term supplementation, without consistent disease-modifying effects [61, 62, 185]. RCTs in schizophrenia and ALS have not demonstrated consistent benefits on core symptoms or disease progression [172, 186, 187].

Taken together, triangulated evidence does not support a broad causal role for vitamin D supplementation in the prevention or treatment of neuropsychiatric or neurodegenerative disorders. Observed benefits are small (e.g. depressive symptoms, select PD functional endpoints), context-specific, and largely confined to individuals with low baseline 25(OH)D and with regular daily dosing, indicating that vitamin D should not be considered a primary disease-modifying therapy.

#### Vitamin D and Respiratory Infections, COVID-19 and long COVID

Respiratory infections constitute a major cause of global morbidity and mortality. Seasonal influenza affects approximately one billion individuals annually, with 3–5 million severe cases worldwide [188], while the COVID-19 pandemic has resulted in over 770 million confirmed cases and more than 7 million deaths globally [189]. In addition, approximately 10–20% of COVID-19 survivors develop post-acute sequelae (long COVID), representing a growing public health burden [190, 191].

Vitamin D plays a role in host defense against respiratory pathogens through the modulation of innate and adaptive immune responses [192]. Respiratory epithelial cells and immune cells express the VDR and 1α-hydroxylase, enabling local activation of vitamin D within the respiratory tract. Vitamin D enhances antimicrobial peptide production, including cathelicidin (LL-37) and defensins, strengthens epithelial barrier integrity, and suppresses excessive pro-inflammatory responses [193]. In SARS-CoV-2 models, vitamin D attenuates pulmonary inflammation and modulates the renin–angiotensin system by suppressing angiotensin II activity and enhancing ACE-2/angiotensin-(1–7) signaling, thereby reducing oxidative stress and lung injury [194].

Observational studies and meta-analyses consistently report associations between low circulating 25(OH)D concentrations and increased risk and severity of acute respiratory infections (ARI), including influenza and community-acquired pneumonia [195–198]. Low vitamin D status has also been associated with higher incidence, severity, and mortality of COVID-19 in both adult and pediatric populations [199, 200]. Several observational studies further suggest an association between low 25(OH)D concentrations and long COVID outcomes. Evidence regarding long COVID is emerging, with some studies reporting lower vitamin D levels among individuals with persistent symptoms and adverse outcomes while others show no clear association [201–208].

MRS have not demonstrated a consistent causal association between genetically determined 25(OH)D concentrations and susceptibility to COVID-19 infection, hospitalization, or disease severity [209–213]. These findings suggest that lifelong vitamin D status may not exert a strong causal effect on COVID-19 outcomes, although non-linear or context-specific effects cannot be excluded [211].

Evidence from supplementation trials is mixed. RCTs and observational studies support a role for vitamin D supplementation in reducing influenza incidence and severity [214]. The landmark individual-participant meta-analysis of 25 RCTs (> 10,000 participants of all ages) demonstrated protection against ARI (OR 0.88; 95% CI 0.81–0.86), particularly with daily or weekly dosing and baseline deficiency [215]. On the contrary, the CORONAVIT trial in the UK (*n* = 6,200) found no reduction in ARI or COVID-19 incidence with 800–3,200 IU/day vitamin D3 supplementation in vitamin D–insufficient adults (25(OH)D < 75 nmol/L) [216], while recent meta-analyses show no overall ARI risk reduction [217, 218]. For COVID-19 treatment, many recent meta-analyses of RCTs found reduced mortality (RR ranging from 0.56 to 0.76) and ICU admissions (RR range 0.32–0.73) in supplemented versus placebo groups, though study heterogeneity and variable dosing limit certainty [219–224]. Evidence for long COVID remains preliminary. Two small RCTs showed improved symptoms after receiving vitamin D3 for 2–6 months [225, 226], particularly in the number of long COVID symptoms, and inflammatory and gut biomarkers (sTNF-RI, sCD163, fungal translocation marker (1,3)-β-d-glucan) when combined with vitamin K2 [225], and in fatigue, anxiety and cognitive symptoms with high-dose supplementation (60,000 IU of vitamin D weekly) compared to placebo [226].

Overall, experimental and observational evidence supports a role for vitamin D in respiratory immune defense, with deficiency being consistently associated with increased risk and severity of ARIs, including influenza and COVID-19. Clinical trial data indicate that vitamin D supplementation may be beneficial mainly in vitamin D–deficient individuals, particularly with daily dosing regimens, while evidence for influenza prevention and long COVID remains inconclusive. Vitamin D should not be regarded as a standalone antiviral therapy, although maintaining serum 25(OH)D concentrations ≥ 50 nmol/L is advisable for optimal immune competence (Table 3).

**Table 3:** List of some meta-analyses and major studies on circulating 25OHD levels, vitamin D supplementation, COVID-19 and long COVID

Meta-analyses (author, year)	Studies/chronic period	Participants	Key findings
Circulating 25OHD levels and COVID-19 and long COVID			
*Meta-analyses of MRS and major MRS*			
Alcalá-Santiago et al., 2025 [213]	Meta-analysis of 8 MRS	417,580 participants GWAS source: UKB, HGI	-No significant causal association between 25(OH)D and COVID-19 (8 MRS). -Only one study showed a correlation between the vitamin D related SNPs and the COVID-19 infection trait (*p* = 0.013). -Pooled estimates confirmed that 25(OH)D was not significantly associated with the risk for infection, hospitalization and severity of COVID-19 (3 MRS) -Only 2 studies accounted for genetic variants associated with vitamin D deficiency and they did not find any significant association with COVID-19 risk or disease outcomes * None of the MRS took free vitamin D into consideration * High variability in study design
Luo et al., 2022 [212]	Systematic review of 50 MRS	Mostly participants of European ancestry Source: UKB and COVID-10 HGI	No strong genetic evidence supporting the role of vitamin D in COVID-19 risk.
Li et al., 2021 [211]	MRS Study	417,342 participants: 1,746 COVID-19 cases and 399 deaths Source: UKB	-No significant associations between plasma 25(OHD concentration (measured at recruitment, on average 11 years ago) and COVID-19 risk or severity after adjustment for confounders. - MR sensitivity analyses indicated a potential causal effect, although the main MRS showed that genetically predicted vitamin D levels were not causally associated with COVID-19 risk (OR 0.77, 95% CI 0.55–1.11, *p* = 0.160). -Analysis of MR-PRESSO suggested a potential causal effect (OR 0.80, 95% CI 0.66–0.98, *p* = 0.03). -Greater protective effect of genetically predicted vitamin D levels when ambient UVB radiation is stronger. -Ambient UVB was strongly and inversely associated with COVID-19 hospitalization and death.
Patchen et al., 2021 [209]	Two-sample MR Study	17,965 COVID-19 cases 1,370,547 controls Primarily of European ancestry (> 75%) Source: UKB, COVID-10 HGI and the SUNLIGHT Consortium	-No evidence for associations of genetically predicted long- term vitamin D levels with risk and severity of COVID-19 infection. -Long-term usual vitamin D nutritional status does not have a causal effect on susceptibility to COVID-19 infection and its severity.
Butler-Laporte et al., 2021 [210]	Two-sample MR Study	GWAS: 443,734 participants Outcome GWASs: 14,134 COVID-19 cases and 1,284,876 controls from 11 countries Primarily of European ancestry Source: UKB, COVID-10 HGI	-Genetically increased 25(OH)D levels by 1 SD on the logarithmic scale had no significant association with COVID-19 susceptibility (OR 0.95, 95% CI 0.84–1.08, *p* = 0.44), hospitalization (OR 1.09, 95% CI 0.89–1.33, *p* = 0.41), and severe disease (OR 0.97, 95% CI 0.77–1.22, *p* = 0.77). - Sensitivity analyses after removal of variants at risk of horizontal pleiotropy showed similar results
*Meta-analyses of observational studies and major studies*			
Mousavi et al., 2025 [227]	Meta-analysis of 12 case-control studies	743 cases 2,863 controls Children 0–18 years old from Turkey and Egypt	-Vitamin D status was significantly associated with COVID-19 in children. -↓ Vitamin D significantly lower in children with COVID-19 -Vitamin D deficiency was significantly associated with COVID-19 severity in children.
Wang et al., 2024 [228]	Meta-analysis of 9 studies (cohort studies, case–control studies and case series)	1,262 children	-Vitamin D insufficiency (serum 25(OH)D < 30 ng/mL or 75 nmol/L) was more common in children with COVID-19 than healthy children (OR 4.86, 95% CI 2.56–9.26). -Vitamin D insufficiency was associated with a higher risk for severe COVID-19 (OR 4.73, 95% CI 1.39–16.11) and lower odds for asymptomatic illness (OR 0.38, 95% CI 0.38–0.81).
Szarpak, 2023 [203]	Meta-analysis of 7 studies	1,920 pregnant women of any gestational age: −955 COVID-19 cases −965 controls	-No significant difference in serum 25(OH)D levels between cases and controls. -Vitamin D was strongly correlated with the severity of COVID-19 during pregnancy.
Bräunlich et al., 2025 [208]	Observational study	148 PCS cases Historical non-PCS controls (data from 2019 to 2022)	-Vitamin D was significantly lower in PCS cases (long COVID) compared to controls
Krčmová et al., 2024 [201]	Prospective observational study	164 adult participants: −108 patients with COVID-19 (omicron and delta variant) (22 patients were followed for 9 months, and 14 had PCS) −56 controls	-↓ serum 25(OH)D at admission in cases than controls. - serum 25(OH)D at admission did not significantly differ between COVID-19 survivors and non-survivors. -No differences in serum 25(OH)D between patients with or without PCS (long COVID).
Wu et al., 2024 [202]	Retrospective study	16,600 COVID-19 cases: −8,300 with VDD (vitamin D < 20 ng/mL) −8,300 controls (vitamin D ≥ 20 ng/mL) Source: TriNetX research network	The VDD group had a higher risk of all-cause ED visits (HR 1.114, 95% CI 1.012–1.226), all-cause hospitalization (HR 1.230, 95% CI 1.105–1.369), and all-cause death (HR 1.748, 95% CI 1.047–2.290), but not post-COVID-19 condition (HR 0.980, 95% CI 0.630–1.523).
Vitamin D Supplementation, COVID-19 and long COVID			
Zhu et al., 2025 [219]	Meta-analysis of 10 RCTs	870 patients with COVID-19 The vitamin D dosage ranged from 3,000 IU to 200,000 IU.	-Vitamin D supplementation was associated with a ↓ risk of mortality during follow-up (RR 0.76, 95% CI 0.60–0.97), but subgroup analysis showed no statistically significant effect. -No statistically significant difference between vitamin D supplementation and no supplementation in terms of 28-day mortality, need for mechanical ventilation and ICU admission, length of hospital and ICU stay. * Evidence was of low to moderate quality
Autier et al., 2025 [220]	Meta-analysis of 9 RCTs	1,468 patients with COVID-19: −755 received vitamin D supplementation −713 controls Vitamin D compounds were cholecalciferol (5 trials), calcifediol (2 trials), or calcitriol (1 trial)	-COVID-19 patients taking vitamin D had a substantially decreased risk of admission to ICU (OR 0.61, 95% CI 0.39–0.95). -Summary odds ratio (sOR) was 0.61 (95% CI 0.39–0.95) for all trials, 0.34 (0.13–0.93) for trials including 50 to < 106 patients and 0.88 (0.62–1.24) for trials including 106 to 548 patients (interaction *p* = 0.04). *Strong publication bias affected small randomized trials
Zhang et al., 2024 [221]	Meta-analysis of 16 RCTs and 5 cohort studies	4,553 patients with COVID-19: − 2,164 intervention group −2,389 controls Age 31–93 years Daily doses of Vitamin D: 20 IU to 600,000 IU	-Vitamin D supplementation significantly reduced the mortality rate (RR 0.72, 95% CI 0.54–0.94, *p* = 0.02) and ICU admission rates (RR 0.58, 95% CI 0.38–0.88, *p* = 0.01), particularly when administered continuously with a total dose of less than 100,000 IU over 14 days, and among those with baseline 25OHD < 30 ng/mL. -No significant effects on the length of hospital stay or endotracheal intubation rates, regardless of the dosing regimen or baseline Vitamin D levels.
Adil et al., 2024 [229]	Meta-analysis of 14 RCTs	2,165 adult patients with COVID-19: −1,102 intervention group −1,063 controls Vitamin D supplementation was mostly oral cholecalciferol 5,000 to 500,000 IU	-Vitamin D supplementation significantly reduced ICU admissions and the need for mechanical ventilation compared to placebo. -Vitamin D did not significantly affect mortality, length of hospital and ICU stay, duration of mechanical ventilation, or the need for supplemental oxygen. *High heterogeneity and variation in vitamin D doses
Ghoreshi et al., 2024 [230]	Meta-analysis of 16 RCTs	1,452 adult patients with COVID-19: −766 intervention group −686 controls Vitamin D doses: 2,000 to 500, 000 IU.	-Vitamin D supplementation significantly reduced hospital length of stay. -More pronounced reduction in studies with vitamin D dosages ≤ 10,000 IU and in patients over 60 years old. *Significant heterogeneity for mortality
Kow et al., 2024 [231]	Meta-analysis of 19 RCTs	2,495 patients with COVID-19	Vitamin D supplementation significantly reduced all-cause mortality in COVID-19 patients, especially in severe cases: -pooled OR 0.72, 95% CI 0.53–0.98 -pooled OR 0.57, 95% CI 0.35–0.92, in subgroup analysis for severe COVID-19 cases
Sobczak et al., 2024 [222]	Meta-analysis of 13 RCTs	All ages (1 RCT in children and 1 RCT in > 65 years old)	-Vitamin D3 supplementation was negatively associated with ICU admission (RR 0.73, 95% CI 0.5–0.95, *p* = 0.02) and mortality associated with COVID-19 (RR 0.56, 95% CI 0.34–0.9, *p* = 0.02). -No significant effect on length of hospital and ICU stay, need for oxygen supplementation and overall mortality.
Sartini et al., 2024 [223]	Meta-analysis of 19 studies: 7 RCTs and 12 analytical studies	1,262,235 adult patients with COVID-19 -intervention group: 502,698 -control group: 759,537 5 studies on health care workers Intervention was vitamin D supplementation before COVID-19: - cholecalciferol: 400-5,000 IU daily, or 5600-50,000 IU weekly, or10,000–100,000 IU monthly -calcifediol 250 µg per dose, or 54,000 IU monthly or 0.266 mg/monthly	-Vitamin D supplementation has a protective effect on the incidence of COVID-19: 1) RCT studies: OR 0.403, 95% CI 0.218–0.747 2) analytical studies: OR 0.592, 95% CI 0.476–0.736 -Vitamin D supplementation significantly reduces the risk for ICU admission (OR 0.317, 95% CI 0.147–0.680). * No standardization of the intervention * Many studies did not provide data on the prevalence of vitamin D deficiency/insufficiency in the population at baseline * 7 studies were retrospective
Sinopoli et al., 2024 [224]	Meta-analysis of 15 RCTs	2,516 patients with COVID-19: −2,471 adults (≥ 18) −45 children (1–17 years) 2,036 of them hospitalized Vitamin D dose (cholecalciferol in 11, calcifediol in 3 and calcitriol in 1 RCT): 1,000–60,000 IU daily for 14 days to 4 months or 100,000–600,000 IU once	-The effects of vitamin D in preventing COVID-19 and long-COVID were contrasting. -Multiple administrations of vitamin D were associated with lower mortality (*n* = 7, RR 0.67, 95% CI 0.49–0.91) compared to the single administration subgroup (*n* = 3, RR 1.52, 95% CI 0.91–2.52). *High heterogeneity, small sample size and low-quality trials

CI: confidence interval; ED: emergency department; GWAS: genome-wide association study; HGI: Host Genetics Initiative; HR: hazard ratio; IU: International Units; MR-PRESSO: Mendelian Randomization Pleiotropy Residual Sum and Outlier; MRS: Mendelian Randomization Studies; OR: odds ratio; PCS: Post-CoronaVirus-19 Disease syndrome; RR: risk ratio; SD: standard deviation; SNP: single nucleotide polymorphism; UKB: UK Biobank; UVB: Ultraviolet radiation B; VDD: vitamin D deficiency; 25(OH)D: 25-hydroxyvitamin D`

#### Vitamin D and Respiratory Disorders

Asthma and chronic obstructive pulmonary disease (COPD) are among the most prevalent chronic respiratory diseases globally, whereas idiopathic pulmonary fibrosis (IPF) represents a less common but increasingly recognized cause of respiratory morbidity. The global prevalence of asthma is estimated at approximately 6–11% across age groups, while COPD affects more than 10% of adults aged ≥ 40 years and remains a leading cause of respiratory-related mortality worldwide [232, 233].

Vitamin D signaling in the airway epithelium and smooth muscle modulates steroid responsiveness, Th17/IL-17 pathways, epithelial barrier integrity, and airway remodeling in asthma [234]. In COPD, vitamin D exerts anti-inflammatory and antioxidant effects, modulates innate and adaptive immunity, and may attenuate airway remodeling and fibrosis [235, 236]. In fibrotic pathways, calcitriol suppresses TGF-β–driven fibroblast activation and extracellular matrix accumulation, reducing fibrosis in murine models and human IPF-derived fibroblasts [237]. These findings support anti-inflammatory and anti-fibrotic actions of vitamin D in lung tissue.

Evidence from MRS does not support a causal association between genetically determined circulating 25(OH)D and asthma susceptibility [238]. For COPD, a meta-analysis of 13 studies (3,667 participants) reported that the VDBP rs7041-GT genotype was associated with a 49% lower COPD risk and higher serum 25(OH)D concentrations [239]. Some two-sample MRS suggest an inverse association between genetically predicted 25(OH)D and COPD, whereas no clear causal association has been demonstrated for IPF [240, 241].

Umbrella reviews and meta-analyses of observational studies have reported associations between low serum 25(OH)D and impaired lung function, increased exacerbation frequency, and reduced corticosteroid responsiveness in asthma and COPD [53, 242–244]. In asthma, lower 25(OH)D correlates with higher IgE, eosinophilia, and reduced cathelicidin, supporting in vivo immunomodulatory effects [245]. A meta-analysis of 15 prospective studies with 12,758 participants and 1,795 cases showed a U-shaped association between maternal 25(OH)D and childhood asthma risk, with lowest risk at approximately 70 nmol/L [246]. A UK Biobank data analysis has indicated lowest COPD risk at around 55 nmol/L and poorer survival at low pre-diagnostic levels, consistent with threshold rather than linear effects [247].

In contrast, RCTs and meta-analyses, including Cochrane reviews, show that vitamin D supplementation does not significantly reduce asthma or COPD exacerbations, nor improve symptom control or lung function in unselected populations [243, 248–250]. In COPD, vitamin D supplementation reduces moderate-to-severe exacerbations only in individuals with baseline 25(OH)D < 25 nmol/L, with no benefit at higher baseline concentrations [251]. For IPF, RCT evidence remains limited and insufficient to support clinical benefit [252].

Overall, while mechanistic and preclinical data support plausible anti-inflammatory and anti-fibrotic roles for vitamin D in respiratory diseases, MRS and contemporary RCT evidence do not support population-wide benefits. Potential clinical effects appear confined to individuals with profound vitamin D deficiency, particularly in reducing COPD exacerbations.

#### Vitamin D and Chronic Kidney Disease

Chronic kidney disease (CKD) affects more than 670 million individuals worldwide and is associated with substantial morbidity, mortality, and healthcare burden [253, 254]. Vitamin D deficiency is highly prevalent in CKD, exceeding 80% in advanced stages, owing to reduced renal 1α-hydroxylase activity, impaired synthesis of calcitriol, urinary loss of vitamin D–binding protein, and chronic inflammation [255].

Vitamin D plays a central role in mineral and bone metabolism in CKD and has been implicated in non-skeletal pathways relevant to disease progression. Experimental studies have shown that vitamin D signaling suppresses secondary hyperparathyroidism, modulates the renin–angiotensin system, attenuates inflammation and fibrosis, and preserves podocyte function, suggesting potential reno-protective effects [255]. These mechanisms provide biological plausibility for broader clinical effects beyond mineral metabolism.

Evidence from MRS does not support a robust causal association between genetically determined circulating 25(OH)D concentrations and CKD risk, progression, or decline in estimated glomerular filtration rate (eGFR) [256]. These findings suggest that low vitamin D status may primarily reflect disease severity rather than acting as an upstream causal factor in CKD development.

Meta-analyses of observational data and a recent cohort study have consistently associated low circulating 25(OH)D concentrations with adverse outcomes in CKD, including higher all-cause and cardiovascular mortality, increased albuminuria, and greater risk of cardiovascular events [65, 257]. In patients receiving maintenance dialysis, each 10 ng/mL increase in serum 25(OH)D has been associated with a 20–30% reduction in all-cause and cardiovascular mortality in pooled observational analyses [65].

A recent meta-analysis has indicated that supplementation with vitamin D (cholecalciferol or ergocalciferol) or active vitamin D analogs improves biochemical parameters in CKD, including reductions in PTH and alkaline phosphatase levels [258]. However, no consistent benefit has been demonstrated for hard clinical outcomes, including mortality, cardiovascular events, fracture risk, or CKD progression [258]. Accordingly, current clinical guidelines recommend monitoring and correction of vitamin D deficiency in CKD, targeting serum 25(OH)D above 75 nmol/L, to optimize mineral metabolism, while emphasizing that evidence for benefit on non-skeletal outcomes remains limited [259, 260]. Routine high-dose supplementation or use of active analogs outside established indications is not supported by current evidence.

Overall, triangulated evidence indicates that vitamin D deficiency is highly prevalent in CKD and is associated with adverse clinical outcomes, but causal evidence for disease modification remains weak, and benefits of supplementation appear confined to correction of deficiency and management of CKD-related mineral and bone disorders.

#### Vitamin D and Overall Mortality

All-cause mortality represents a critical integrative outcome reflecting the cumulative effects of chronic disease burden, frailty, and systemic dysregulation. Low circulating 25(OH)D concentrations have been consistently associated with increased all-cause mortality across diverse populations, age groups, and geographic regions.

Observational cohort studies and meta-analyses demonstrate a robust inverse association between serum 25(OH)D concentrations and all-cause mortality, with the highest risk observed at low vitamin D levels [37, 63, 64]. Pooled analyses indicate that individuals in the lowest 25(OH)D categories have approximately a 30–70% higher risk of death compared with those in the highest categories, with a non-linear, reverse J-shaped association [63, 106]. Mortality risk increases steeply below approximately 30–40 nmol/L and plateaus at higher concentrations, suggesting a threshold rather than linear relationship [106]. Comparative analyses show similar inverse associations for total, bioavailable, and free 25(OH)D, indicating no additional predictive value of alternative biomarkers beyond total 25(OH)D [64].

MRS provide mixed evidence. Some analyses support a non-linear causal association between genetically predicted low 25(OH)D concentrations and increased all-cause mortality, with excess risk confined to individuals with marked deficiency [37, 261, 262]. In contrast, one of the largest MRS to date, including 386,406 individuals from four population-based cohorts, found no association between genetically predicted 25(OH)D and all-cause or cause-specific mortality, even across the full range of observed 25(OH)D concentrations [104]. These discrepancies highlight methodological limitations and potential threshold-dependent effects.

RCTs generally report neutral effects of vitamin D supplementation on all-cause mortality in unselected or vitamin D–replete populations. Large primary-prevention trials, including VITAL, D-Health, ViDA, and FIND, showed no reduction in all-cause mortality with supplementation [66, 69, 75, 79]. However, umbrella reviews and meta-analyses of RCTs suggest a modest but consistent reduction in all-cause mortality, particularly with daily vitamin D supplementation (typically 800–4,000 IU/day), and among older adults or individuals with low baseline 25(OH)D concentrations [3, 37, 263]. The ES guideline reports an approximate 4–6% reduction in all-cause mortality in adults aged ≥ 75 years receiving vitamin D supplementation (primarily cholecalciferol, often at standard daily doses), corresponding to a small but clinically meaningful absolute risk reduction (6 fewer deaths per 1000 people) [3]. This effect was consistent across subgroups, including those with low baseline 25(OH)D, and was not significantly modified by sex, calcium co-administration, or care setting (community, nursing homes or hospital clinics). In contrast, no survival benefit was observed in adults aged 50–74 years, and subgroup analyses suggested potential harm with high-dose vitamin D alone, while co-administration with calcium may reduce mortality risk [3].

In summary, triangulated evidence supports a non-linear association between vitamin D status and all-cause mortality, with increased risk concentrated at low 25(OH)D concentrations. Supplementation-associated mortality benefits appear modest, threshold-dependent, and largely confined to older (aged ≥ 75 years) or vitamin D–deficient populations, whereas routine supplementation in vitamin D–replete and younger adults does not confer survival benefit.

## Controversies and Methodological Challenges in Vitamin D Research

Despite decades of investigation, the role of vitamin D in health outcomes remains one of the most debated topics in medicine. Inconsistencies among observational studies, MRS, and RCTs continue to fuel controversy regarding causality, optimal thresholds and supplementation strategies. Observational studies often suggest broad health benefits, whereas RCTs and MRS frequently demonstrate null or weak causal effects, particularly in vitamin D–replete populations [37, 38, 264]. This divergence has led to the interpretation that vitamin D may function, in many contexts, as a biomarker of overall health status rather than a direct causal mediator, although heterogeneity across populations, latitude, baseline vitamin D status, and genetic background complicates generalization [37, 264].

Differences in study design, target populations, dosing regimens, and definitions of vitamin D sufficiency further complicate interpretation. In addition, substantial inter-assay and inter-laboratory variability in 25(OH)D measurement represents an important and often underappreciated source of heterogeneity, with reported between-method differences frequently exceeding 10–20 nmol/L and influencing both prevalence estimates and exposure–outcome associations [8]. Many large vitamin D RCTs were designed following a pharmacologic paradigm, frequently enrolling vitamin D–replete individuals, applying fixed doses, permitting background supplementation in control groups, and relying on intention-to-treat analyses rather than achieved 25(OH)D concentrations, conditions that may underestimate biologically meaningful effects confined to deficiency states [66, 264].

### Limitations of Observational Evidence

Observational data remain valuable for hypothesis generation but cannot establish causality [37, 38]. Large epidemiologic cohorts consistently show inverse associations between serum 25(OH)D and diverse outcomes, including CVD, cancer, metabolic and autoimmune disorders, and overall mortality [37]. However, residual confounding and reverse causation remain major limitations, as low 25(OH)D frequently reflects poor health status, obesity, physical inactivity, or reduced sun exposure rather than causal disease pathways [264]. Lifestyle and socioeconomic factors are difficult to fully adjust for and may inflate observed associations [37, 38]. Furthermore, assay-related variability and incomplete standardization of 25(OH)D measurement may contribute to exposure misclassification, further diluting true associations and increasing heterogeneity across observational analyses [8]. In addition, most epidemiologic studies rely on total circulating 25(OH)D concentrations, whereas free and bioavailable fractions may better reflect tissue-level activity in selected contexts. Variability in VDBP concentrations and genetic polymorphisms may further influence measured total 25(OH)D without necessarily altering intracellular signaling. These factors may partially explain discrepancies between observational associations based on measured total 25(OH)D and findings from genetic or interventional studies.

Interpretation is further complicated by non-linear dose–response relationships. Meta-analyses indicate threshold effects, with risk increasing steeply at serum 25(OH)D concentrations below approximately 25–40 nmol/L and little additional benefit beyond 50–60 nmol/L [63, 106]. Non-linear associations have been reported for all-cause and cardiovascular mortality whereas type 2 diabetes risk appears to decline more linearly with increasing 25(OH)D [63, 106, 265]. Evidence of U-shaped relationships for outcomes such as falls and fractures suggests potential harm at both very low and very high concentrations [266].

### Controversies in MRS

MRS exploit genetic variants involved in vitamin D synthesis, transport, and metabolism (e.g. *GC*,* CYP2R1*,* DHCR7/NADSYN1*) to reduce confounding and reverse causation [30]. These analyses approximate lifelong differences in vitamin D status, but findings remain heterogeneous. Large two-sample MRS involving up to 500,000 participants have generally failed to demonstrate robust causal effects of genetically higher 25(OH)D concentrations on CVD, cancer, T2DM, or all-cause mortality in largely vitamin D–replete populations [30, 34, 104]. In contrast, non-linear MRS have suggested L-shaped associations for mortality, with excess risk confined to low 25(OH)D concentrations, supporting a threshold-dependent model [106].

Discrepant MRS findings likely reflect methodological differences, including instrument strength, ancestry composition, and assumptions of linearity. Genetic instruments typically explain less than 5% of the variance in circulating 25(OH)D, limiting power to detect modest effects [30]. Moreover, most MRS instrument total circulating 25(OH)D and do not account for free or bioavailable fractions or VDBP variability, which may limit interpretation in conditions where binding protein dynamics are altered (e.g. pregnancy, chronic inflammation, or liver disease). Moreover, MRS estimate lifelong exposure and may not capture short-term or adult-onset supplementation effects. Additional concerns include pleiotropy and violation of exclusion-restriction assumptions, as well as the inability of MRS to account for tissue-specific activation of vitamin D or interactions with calcium, magnesium, and PTH that may be critical for extra-skeletal effects [30, 264].

### Methodological and Interpretative Challenges in RCTs

RCTs remain the cornerstone of evidence-based medicine; however, most large vitamin D trials, such as VITAL, ViDA, D-Health, DO-HEALTH and FIND, reported null or minimal benefits on major health outcomes [66, 75, 267]. Key limitations include enrollment of vitamin D–replete participants, fixed-dose regimens that ignore inter-individual variability, heterogeneous dosing schedules, limited adherence, and insufficient duration to assess long-term disease endpoints. Accumulating evidence suggests that daily or weekly supplementation is more physiologically appropriate than large intermittent bolus dosing, which may transiently elevate FGF23 and adversely affect bone outcomes [267]. Importantly, nutrients differ fundamentally from drugs, acting as threshold-dependent modulators within complex biological systems and interacting with co-nutrients and environmental factors such as sunlight exposure [264]. Moreover, a critical distinction in vitamin D research is the difference between correcting deficiency and administering supplementation to individuals who are already vitamin D–replete. Trials designed to restore deficient individuals to physiologic sufficiency test a replacement paradigm, whereas large population-based trials enrolling predominantly replete participants often evaluate pharmacologic supplementation beyond baseline adequacy. Failure to distinguish between these paradigms may contribute to apparent discrepancies between observational associations and neutral trial findings.

Meta-analyses of RCTs demonstrate no overall reduction in all-cause mortality but report a small, consistent reduction in cancer mortality with daily vitamin D supplementation, whereas bolus regimens appear neutral or potentially harmful [264, 267]. These findings underscore that RCT results cannot be generalized without careful consideration of baseline vitamin D status, dosing frequency, and biological context.

## Conclusion

The synthesis of current evidence indicates that vitamin D exerts clear, causal effects in specific pathophysiologic contexts, rather than uniform benefits across all health outcomes. As summarized in Table 4, strong and consistent triangulated evidence supports a causal role for vitamin D in skeletal health, particularly in the prevention and treatment of rickets and osteomalacia, and in fracture risk reduction among institutionalized or deficient individuals in combination with calcium. These outcomes show alignment across observational studies, genetic analyses, and intervention trials, reinforcing the biological necessity of adequate vitamin D for mineral metabolism.

For selected extra-skeletal outcomes, moderate, threshold-dependent benefits are also evident for cancer mortality, selected autoimmune disorders, most convincingly MS, and respiratory infections, including COVID-19, primarily in individuals with baseline deficiency or increased physiological demands such as pregnancy, aging, and chronic illness. These effects may be explained by the regulatory roles of vitamin D in immune modulation, inflammation, and cellular homeostasis rather than direct disease-specific mechanisms.

In contrast, associations with cardiovascular, metabolic, and neuropsychiatric outcomes appear weak or largely non-causal, as observational signals are not consistently reproduced in genetic or interventional analyses, suggesting residual confounding by lifestyle factors, adiposity, and overall health status. For T2DM, individual participant data meta-analyses of RCTs in prediabetic populations show a 15% reduction in diabetes risk with vitamin D supplementation, with greater benefit at higher achieved 25(OH)D levels [129].

All-cause mortality occupies an intermediate position within the triangulated framework, with excess risk consistently observed at concentrations below approximately 30–40 nmol/L and limited additional benefit beyond sufficiency. Observational studies consistently demonstrate a strong inverse association between low 25(OH)D concentrations and mortality risk, while genetic and interventional evidence supports a non-linear, threshold-dependent relationship, with excess risk concentrated on low vitamin D levels and limited benefit beyond sufficiency. Supplementation-associated reductions in all-cause mortality are modest and largely confined to older adults or vitamin D-deficient populations.

Taken together, the triangulated synthesis presented in Table 4 may reconcile longstanding inconsistencies in vitamin D research by reframing vitamin D as a context-dependent determinant of health. These findings argue against indiscriminate population-wide pharmacologic supplementation and instead support targeted strategies focused on the identification and physiologic correction of deficiency. Therefore, vitamin D should be regarded neither as a universal panacea nor as a negligible nutrient, but as a hormone whose clinical relevance emerges when deficiency compromises skeletal integrity and immune-regulatory functions.

**Table 4:** Triangulation of evidence linking low vitamin d status with health outcomes

Disease/Outcome	Observational Evidence	MR evidence	RCT Evidence	Triangulation Strength	Summary Comment
Skeletal (fractures, BMD, rickets, osteomalacia)	✓ ✓ ✓ Strong inverse association	✓ - Causal for rickets/osteomalacia --Weak for BMD/fractures except in severe deficiency -Confirms threshold effects	✓ Clear benefit with supplementation in deficiency -Prevention/treatment of rickets/osteomalacia -↓ fracture in deficient/institutionalized individuals mainly and when combined with calcium	**-High for rickets/osteomalacia -Moderate for fractures/BMD**	-Causality established for rickets/osteomalacia -fracture benefit limited to deficient/high-risk groups and when combined to calcium
Cardiovascular disease	✓ Inverse association for CVD risk, including stroke and PAD	-No causal link **-**Weak evidence for CVD (HF, dyslipidemia)	-Null results in large RCTs for CVD, MI, HF, stroke or MACE - No benefit in primary prevention	**Weak**	- No support for causality -Observational links potentially confounded by comorbidities and lifestyle
Cancer -incidence -mortality	✓ Inverse associations for cancer risk ✓ Consistent inverse association for mortality -Strongest and most consistent findings for colorectal, lung, and breast cancers -Less consistent or null associations for prostate and other cancer types	Generally null for incidence, especially for colorectal, breast, lung, prostate, pancreatic, ovarian cancers and neuroblastoma Null associations, but overall limited MR data	Mostly null; no prevention in replete groups Summary RR ~ 0.98, 95% CI: 0.94–1.02 ✓ possible ↓ in cancer mortality in deficient populations and with daily dosing regimens Summary RR ~ 0.88, 95% CI: 0.80–0.96 -modest survival benefit	**Limited–Moderate** **Moderate**	-Observational signals not confirmed by MR or RCTs for cancer prevention -Benefit confined to mortality in deficient individuals
Autoimmune disorders	✓ Inverse associations (especially MS, weaker for psoriasis, SLE and RA)	✓ Causal for MS, suggestive for psoriasis, less consistent with AT, SLE and RA	✓ ↓ Autoimmune disease incidence in supplementation trials (−22%), particularly in deficiency - No clear benefit except possible ↓ in MS risk/relapse	**-Moderate–Emerging for MS** **-Weak for others**	Convergent evidence; strongest for MS and less consistent for psoriasis -other autoimmune links less robust
Metabolic disorders (T2DM, T1DM, dyslipidemia, MASLD)	✓ Inverse associations with T2DM, T1DM, dyslipidemia, Mets, MASLD	No causal effect for T2DM/T1DM -Suggestive for MASLD/NAFLD	Modest improvements especially in deficiency **-** Overall, no effect on T2DM, T1DM and MASLD prevention; null in large RCTs -↓ progression from prediabetes to diabetes -small improvements in glycemic markers in diabetic and prediabetic subjects	**Limited**	-No prevention effect. Benefits confined to deficient populations - Observational associations not supported by genetic or trial evidence
Infectious diseases (ARI, flu, COVID-19) and long COVID	✓ -Deficiency linked to ↑ ARI, pneumonia and mortality, influenza and COVID-19, including severity and mortality -Suggestive for LC and its outcomes (symptoms burden, severity, delayed recovery)	-Null for susceptibility to respiratory infections overall; possible effect under severe deficiency -No clear causal effect for COVID-19 and its severity	✓ -mixed results -modest ↓ ARI and COVID-19 severity with daily dosing and baseline deficiency -preliminary evidence suggestive for LC symptoms improvements (small RCTs)	**Moderate**	-Benefit in ARI mainly under deficiency - No robust effect for COVID-19 or influenza -Limited data for LC
Respiratory diseases -COPD -asthma -IPF	✓ Low 25(OH)D linked to severity/exacerbations ✓ Lower 25(OH)D linked to poor control and impaired lung function -U shaped association between maternal blood 25(OH)D and childhood asthma (↓↓ risk at ~ 70 nmol/L) ✓ Deficiency common; correlates with worse outcomes	Limited MR data suggestive for an inverse association No causal signal MRS show no clear causal effect	✓ ↓ Exacerbations only if baseline < 25 nmol/L Mixed; ↓ exacerbations in selected subgroups Limited RCT data	**Moderate** **Limited–Moderate** **Weak**	-Benefit limited to severely deficient patients -Inconsistent evidence; possible benefit in deficient or pediatric populations -Mechanistically plausible but unproven clinically
Neuropsychiatric/neurodegenerative disorders	✓ Linked to cognitive decline, depression -↓ 25(OH)D associated with all-cause dementia, AD, ALS, PD and schizophrenia risks -↓neonatal 25(OH)D associated with later schizophrenia risk	No genetic causality overall, especially for depression and PD **-** Mixed evidence, suggestive for all-cause dementia, AD, schizophrenia and ALS; not robust	- No effect in RCTs -Mixed results in deficiency -Small improvements in depressive symptoms and motor scales in PD -No benefit for ALS progression -No consistent benefit for schizophrenia (core psychotic symptoms)	**Weak**	Likely indirect or non-causal association
CKD	✓ Low 25(OH)D and 1,25(OH)₂D correlate with CKD progression (mortality, albuminuria, CV risk)	No clear causal effect	✓ Improves PTH and phosphate, not mortality **-** No benefit in RCTs (mortality, eGFR, CV events, fracture risk)	**Weak**	-Strong physiologic basis; limited outcome-level proof -No clear evidence for causality or benefit
All-cause mortality	✓ Strong inverse association	✓ Nonlinear causal effect (< 40 nmol/L)	✓ -↓ Mortality in elderly (> 75y) or deficient populations -Null in replete populations	**Moderate–Strong**	Threshold-dependent effect; benefits primarily in deficiency and adults over 75y

✓ qualitative indicator of supportive evidence; 25(OH)D: 25-hydroxyvitamin D; 1,25(OH)₂D: 1,25-dihydroxyvitamin D (calcitriol); ARI: acute respiratory infection; AD: Alzheimer’s Disease; AT: autoimmune thyroiditis; BMD: bone mineral density; Ca: calcium; CKD: chronic kidney disease; CKD–MBD: chronic kidney disease–mineral and bone disorder; COPD: chronic obstructive pulmonary disease; COVID-19: coronavirus disease 2019; eGFR: estimated Glomerular Filtration Rate; FGF23: fibroblast growth factor 23; IPF: idiopathic pulmonary fibrosis; LC: long COVID; MACE: Major Adverse Cardiac Events; MASLD: metabolic dysfunction–associated steatotic liver disease; Mets: metabolic syndrome; MR: Mendelian randomization; MS: multiple sclerosis; PAD: peripheral artery disease; PTH: parathyroid hormone; RA: Rheumatoid Arthritis; RCT: randomized controlled trial; SLE: Systemic lupus erythematosus; T1D: type 1 diabetes; T2D: type 2 diabetes; VD: vitamin D

## Key References

- Liu D, Meng X, Tian Q, Cao W, Fan X, Wu L, Song M, Meng Q, Wang W, Wang Y. Vitamin D and Multiple Health Outcomes: An Umbrella Review of Observational Studies, Randomized Controlled Trials, and Mendelian Randomization Studies. Adv Nutr. 2022 ;13(4):1044–1062. doi: 10.1093/advances/nmab142.
  - ○ This is an Umbrella Review of Observational Studies, Randomized Controlled Trials, and Mendelian Randomization Studies regarding vitamin D and multiple health outcomes.
- Fang A, Zhao Y, Yang P, Zhang X, Giovannucci EL. Vitamin D and human health: evidence from Mendelian randomization studies. Eur J Epidemiol. 2024;39(5):467–490. doi: 10.1007/s10654-023-01075-4.
  - ○ Evidence from Mendelian randomization studies provides strong support for a causal link between vitamin D and the development of multiple sclerosis.
- Demay MB, Pittas, (A) G., Bikle, D. D., Diab, D. L., Kiely, M. E., Lazaretti-Castro, M., et al. Correction to Vitamin D for the Prevention of Disease: An Endocrine Society Clinical Practice Guideline. J Clin Endocrinol Metab. 2025; 110(3):e916. 10.1210/clinem/dgae854.
  - ○ The Endocrine Society panel recommends empiric vitamin D supplementation for individuals aged 1–18 years, adults over 75 years, pregnant individuals, and those with high-risk prediabetes.
- Zhu L, Zhang Y, Li X, Zou X, Bing P, Qi M, He (B) Vitamin D supplementation for managing COVID-19 in patients with vitamin D deficiency: a systematic review and meta-analysis of randomised controlled trials. BMJ Open. 2025;15(3):e091903. doi: 10.1136/bmjopen-2024-091903.
  - ○ In this meta-analysis of RCTs, vitamin D supplementation reduced overall mortality during follow-up in vitamin D–deficient COVID-19 patients but had no effect on short-term mortality, ICU or ventilation needs, or length of hospitalization.

## Data Availability

No datasets were generated or analysed during the current study.

## Abbreviations

1,25(OH)₂D: 1,25-dihydroxyvitamin D (calcitriol)
25(OH)D: 25-hydroxyvitamin D
AD: Alzheimer’s disease
AF: atrial fibrillation
AILD: autoimmune liver disease
AIH: autoimmune hepatitis
ALS: amyotrophic lateral sclerosis
AMSTAR-2: A Measurement Tool to Assess Systematic Reviews
ARI: acute respiratory infection
BCC: basal cell carcinoma
BMD: bone mineral density
BMI: body mass index
BP: blood pressure
CC: colorectal cancer
CHD: congenital heart disease
CKD: chronic kidney disease
CKD–MBD: chronic kidney disease–mineral and bone disorder
COPD: chronic obstructive pulmonary disease
COVID-19: coronavirus disease 2019
CrI: credible interval
CVD: cardiovascular disease
CYP2R1: cytochrome P450 2R1 (vitamin D 25-hydroxylase)
CYP27A1: cytochrome P450 27A1
CYP27B1: 1α-hydroxylase
CYP24A1: cytochrome P450 24-hydroxylase
DBP: vitamin D–binding protein
FGF23: fibroblast growth factor 23
HDL-C: high-density lipoprotein cholesterol
HOMA-IR: homeostatic model assessment of insulin resistance
HR: hazard ratio
IPF: idiopathic pulmonary fibrosis
IU: international units
LDL-C: low-density lipoprotein cholesterol
MASLD: metabolic dysfunction–associated steatotic liver disease
MACE: major adverse cardiovascular events
MI: myocardial infarction
MRS: Mendelian randomization Study
MS: multiple sclerosis
NAM: National Academy of Medicine
NHANES: National Health and Nutrition Examination Survey
OR: odds ratio
PAD: peripheral artery disease
PD: Parkinson’s disease
PE: preeclampsia
PTH: parathyroid hormone
RAS: renin–angiotensin system
RCT: randomized controlled trial
RR: relative risk
RXR: retinoid X receptor
SBP: systolic blood pressure
SD: standard deviation
SLE: systemic lupus erythematosus
SNP: single-nucleotide polymorphism
TG: triglycerides
T1D: type 1 diabetes
T2D: type 2 diabetes
UL: upper intake level
UVB: ultraviolet B
VDBP: vitamin D–binding protein
VDR: vitamin D receptor
VDSP: Vitamin D Standardization Program
WHR: waist–hip ratio
